# Immunoautophagy-Related Long Noncoding RNA (IAR-lncRNA) Signature Predicts Survival in Hepatocellular Carcinoma

**DOI:** 10.3390/biology10121301

**Published:** 2021-12-09

**Authors:** Yulu Wang, Fangfang Ge, Amit Sharma, Oliver Rudan, Maria F. Setiawan, Maria A. Gonzalez-Carmona, Miroslaw T. Kornek, Christian P. Strassburg, Matthias Schmid, Ingo G. H. Schmidt-Wolf

**Affiliations:** 1 Center for Integrated Oncology (CIO), Department of Integrated Oncology, University Hospital of Bonn, 53127 Bonn, Germany; Yulu.Wang@ukbonn.de (Y.W.); Fangfang.Ge@ukbonn.de (F.G.); Amit.Sharma@ukbonn.de (A.S.); oliver.rudan@ukbonn.de (O.R.); Maria_fitria.setiawan@ukbonn.de (M.F.S.); 2 Department of Neurosurgery, University Hospital of Bonn, 53127 Bonn, Germany; 3 Department of Internal Medicine I, University Hospital of Bonn, 53127 Bonn, Germany; maria.gonzalez-carmona@ukbonn.de (M.A.G.-C.); Miroslaw_theodor.kornek@ukbonn.de (M.T.K.); christian.strassburg@ukbonn.de (C.P.S.); 4 Institute of Medical Biometry, Informatics and Epidemiology, University Hospital of Bonn, 53127 Bonn, Germany; matthias.schmid@ukbonn.de

**Keywords:** liver cancer, hepatocellular carcinoma, lncRNAs, autophagy, biomarker, kyoto encyclopedia of genes and genomes, prognosis, signature, immune genes

## Abstract

**Simple Summary:**

Hepatocellular carcinoma (HCC) is the most common type of primary liver cancer, which is more prevalent in adults. Herein, we established the first immuno-autophagy-related long non-coding RNA (IARlncRNA) signature displaying a prognostic ability among HCC patient groups.

**Abstract:**

Background: The dysregulation of autophagy and immunological processes has been linked to various pathophysiological conditions, including cancer. Most notably, their particular involvement in hepatocellular carcinoma (HCC) is becoming increasingly evident. This has led to the possibility of developing a prognostic signature based on immuno-autophagy-related (IAR) genes. Given that long non-coding RNAs (lncRNAs) also play a special role in HCC, a combined signature utilizing IAR genes and HCC-associated long noncoding RNAs (as IARlncRNA) may potentially help in the clinical scenario. Method: We used Pearson correlation analysis, Kaplan–Meier survival curves, univariate and multivariate Cox regression, and ROC curves to generate and validate a prognostic immuno-autophagy-related long non-coding RNA (IARlncRNA) signature. The Chi-squared test was utilized to investigate the correlation between the obtained signature and the clinical characteristics. CIBERSORT algorithms and the Wilcoxon rank sum test were applied to investigate the correlation between signature and infiltrating immune cells. GO and KEGG analyses were performed to derived signature-dependent pathways. Results: Herein, we build an IAR-lncRNA signature (as first in the literature) and demonstrate its prognostic ability in hepatocellular carcinoma. Primarily, we identified three IARlncRNAs (MIR210HG, AC099850.3 and CYTOR) as unfavorable prognostic determinants. The obtained signature predicted the high-risk HCC group with shorter overall survival, and was further associated with clinical characteristics such as tumor grade (t = 10.918, *p* = 0.001). Additionally, several infiltrating immune cells showed varied fractions between the low-risk group and the high-risk HCC groups in association with the obtained signature. In addition, pathways analysis described by the signature clearly distinguishes both risk groups in HCC. Conclusions: The immuno-autophagy-related long non-coding RNA (IARlncRNA) signature we established exhibits a prognostic ability in hepatocellular carcinoma. To our knowledge, this is the first attempt in the literature to combine three determinants (immune, autophagy and LnRNAs), thus requiring molecular validation of this obtained signature in clinical samples.

## 1. Introduction

Autophagy as a conserved process captures and degrades intracellular components primarily to maintain metabolism and cellular homeostasis. Dysregulation of this process has been linked to several pathophysiological conditions, such as cancer and neurodegenerative diseases [1,2]. Particularly in hepatocellular carcinoma (HCC), autophagy has been shown to play a role by promoting the metastatic colonization of HCC cells [3].

HCC, the most common malignancy of the liver, is currently the fourth leading cause of cancer-related death worldwide [4]. Primary risk factors for the development of HCC include chronic liver disease and cirrhosis, most of which are caused by chronic viral hepatitis (B + C) and excessive alcohol consumption. Several genetic and epigenetic factors have also been implicated in the molecular pathogenesis of HCC [5]. Considering the overlap of mutational pathways in cancers [6], studies have also prompted the analysis of the prognostic potential of certain genes across the spectrum of multiple cancers, including HCC [7]. Likewise, the relative contribution of autophagy in HCC is becoming increasingly apparent; for instance, Wu et al. showed that autophagic degradation machinery and the cell-cycle regulator cyclin D1 are linked to HCC tumorigenesis [8]. It has also been discussed that activation of autophagy decreases the expression of oncogenic microRNA-224, and thus impedes tumorigenesis in hepatitis B virus-related HCC [9]. Of interest, several compounds have been shown to exert antitumor effects in liver cancer via autophagy [10,11,12]. In the context of autophagy-related genes (ATG), lower expression was previously observed in HCC, which was predicted to contribute to tumor growth and the poor prognosis of the disease [13,14]. Of interest, there have been few recent attempts to identify a prognostic signature of ATGs in HCC [15,16]. Besides this, immunoautophagy-related genes (IARGs) were also recently evaluated for their potential prognostic significance in HCC patients [17]. Considering that long non-coding RNAs (lncRNAs) also play a special role in cancer, their ability to regulate tumor growth by modulating autophagy in liver, bladder, and pancreatic cancers has already been implicated [18,19]. In HCC, a study discussed the potential involvement of lncRNA HULC (highly upregulated in liver cancer) in the autophagy and chemoresistance of HCC cells [20]. Similarly, the lncRNA SNHG1 has been shown to induce resistance to the drug sorafenib in HCC through activation of the Akt pathway [21]. Recently, the prognostic value of an autophagy-related lncRNA signature in HCC has been discussed [22].

Considering this plethora of literature, we have attempted to combine immune-, autophagy, and noncoding RNAs to generate immunoautophagy-related long noncoding RNA (IAR-lncRNA). Herein, we build an IAR-lncRNA signature (first in the literature) and demonstrate its prognostic ability in hepatocellular carcinoma.

## 2. Materials and Methods

### 2.1. Gene Expression Data and Clinicopathological Characteristics

Gene expression data (workflow type: HTSeq—FPKM) and associated clinical information of patients with hepatocellular carcinoma of the liver (HCC) were downloaded from UCSC Xena (https://xena.ucsc.edu/, accessed on 22 October 2021). The reference database was the GDC TCGA Liver Cancer (LIHC) dataset, which contains 374 tumor samples with comprehensive gene expression data. Of these, 371 samples were from primary tumors (mainly used in this study), and the remaining 3 samples were from recurrent tumors (3 samples from 2 patients), which were excluded from the analysis. Only 365 samples have both gene expression data and survival data (survival time and survival status). Based on the available clinical characteristics, only 163 samples were further processed for the clinical comparisons. In total, 210 genes involved in autophagy were retrieved from the Human Autophagy Database (HADb, http://autophagy.lu/clustering/index.html, accessed on 22 May 2021). A total of 1344 immune-related genes were retrieved from Immport Shared Data (https://www.immport.org/shared/home, accessed on 27 June 2021). We focused our analysis on 371 HCC samples, excluding recurrent samples due to their peculiar clinical/biological characteristics. Log2(FPKM + 1) gene expression data were applied to obtain AR genes, IR genes and lncRNAs. Due to the sizes of genes and lncRNAs, the average gene expression (log2(FPKM + 1)) of AR genes and IR genes (no more than 0) and lncRNAs (no more than 0.5) was excluded. Log2 was further applied for the gene expression data (log2(FPKM + 1)) in order to obtain fitting normalized distribution. Since lncRNAs were expressed at relatively low levels, the correlation of gene (AR and IR) expression (Log2(log2(FPKM + 1) + 1)) and lncRNA expression (log2(FPKM + 1)) was used to establish AR- and IR-related lncRNAs. LncRNA expression (Log2(log2(FPKM + 1) + 1)) data were subsequently used in statistical analyses.

### 2.2. Development of the Prognostic Immuno-Autophagy-Related lncRNAs Signature

Univariate Cox regressions were applied to select survival-related autophagy genes and immune genes, which were based on *p*-values < 0.01. Then the correlation between lncRNAs and survival-related autophagy genes was determined by Pearson correlation analysis. LncRNAs with correlation coefficients |R| > 0.4 and *p* values < 0.01 were considered autophagy-related. The correlation between lncRNAs and survival-related immune genes was determined by Pearson correlation analysis. LncRNAs with correlation coefficients |R| > 0.6 and *p* values < 0.01 were defined as immune-related. Thus, we obtained autophagy-related lncRNAs (ARlncRNAs) and immune-related lncRNAs (IRlncRNAs) for the further steps. Next, we determined the lncRNA was associated with immunoautophagy (IARlncRNA) if the lncRNA belonged to both ARlncRNAs and IRlncRNAs concurrently. Then, univariate Cox regression was performed to select survival-related IARlncRNA. Subsequently, multivariate Cox regression analysis was performed based on the lowest Akaike information criterion (AIC) to determine the optimal prognostic signature. Risk scores were calculated using the following formula: (βgene 1 × expgene 1) + (βgene 2 × expgene 2) + --- + (βgene *n* × expgene *n*). Here, expgene represents the expression of lncRNA. Of note, the cutoff value for the high-risk group and the low-risk group was the median risk score. The differential expressions of the lncRNAs in signature between high- and low-risk groups were assessed by Wilcoxon rank sum test.

### 2.3. Prognostic Ability of Immuno-Autophagy-Related lncRNAs Signature

The Kaplan–Meier survival curve was applied to investigate the survival rate between high-risk and low-risk groups, and *p* < 0.05 was considered as a significant difference. Subsequently, an ROC curve was performed to test the predicting value of the signature. Univariate Cox regression and multivariate Cox regression were used to assess the independent ability of the signature, primarily based on *p* < 0.05 when clinical features (age, gender, Child–Pugh classification, AFP, fibrosis, grade and stage) were considered.

### 2.4. Correlation between Immune Cells and Signature

CIBERSORT analysis was performed to explore the percentages of 22 immune cells in each patient. Wilcoxon rank-sum test was used to determine the varying of immune cells in low- and high-risk groups (*p* < 0.05).

### 2.5. GO and KEGG Analysis

Differential genes were found between the low-risk group and the high-risk group based on log2 fold change (logFC) > 1 and false discovery rate (FDR) < 0.05 using the Wilcoxon rank sum test. Subsequently, these genes were included in GO and KEGG analyses using the R package “clusterProfiler” to explore pathways, which were selected with a *q* value < 0.05.

### 2.6. Statistical Analysis

Pearson correlation analysis, Chi-squared test, Wilcoxon rank sum test, Cox regression, Kaplan–Meier curves, survival status, heat map, ROC curve, cibersort algorithm, GO analysis and KEGG analysis were performed using R software. The coexpression network between genes (ARgenes and IRgenes) along with an lncRNA coexpression network was illustrated using CYTOSCAPE software.

## 3. Results

### 3.1. Correlating Autophagy-Related Genes and Immune-Related Genes with lncRNAs

We first derived autophagy-related genes from the Human Autophagy Database (HADb, http://autophagy.lu/clustering/index.html, accessed on 22 May 2021) and immune-related genes from the Immport Shared Data (https://www.immport.org/shared/home, accessed on 27 June 2021). Subsequently, the gene expression datasets of LIHC (GDC TCGA Liver Cancer (LIHC)) were downloaded from the UCSC Xena. Next, we extracted the lncRNA genes, autophagy-related (AR) genes and immune-related (IR) genes corresponding to HCC from the TCGA data. First, univariate Cox regressions were performed to select survival-related AR genes and IR genes. Subsequently, Pearson correlation was used to confirm the correlation between autophagic genes and lncRNA (|R| > 0.4 and *p*-value < 0.01), in addition to the correlation between immune-related genes and lncRNA (|R| > 0.6 and *p*-value < 0.01). Using these parameters, a total of 244 ARlncRNAs (Supplementary File S1) and 36 IRlncRNAs (Supplementary File S2) was identified. When combined, the ARlncRNAs and IRlncRNAs yielded 36 IARlncRNAs. The overview of the complete strategy is shown in a flowchart (Figure S1).

### 3.2. A Signature Involving 3 Immuno-Autophagy-Related lncRNAs with Prognostic Potential

**Figure 1:**
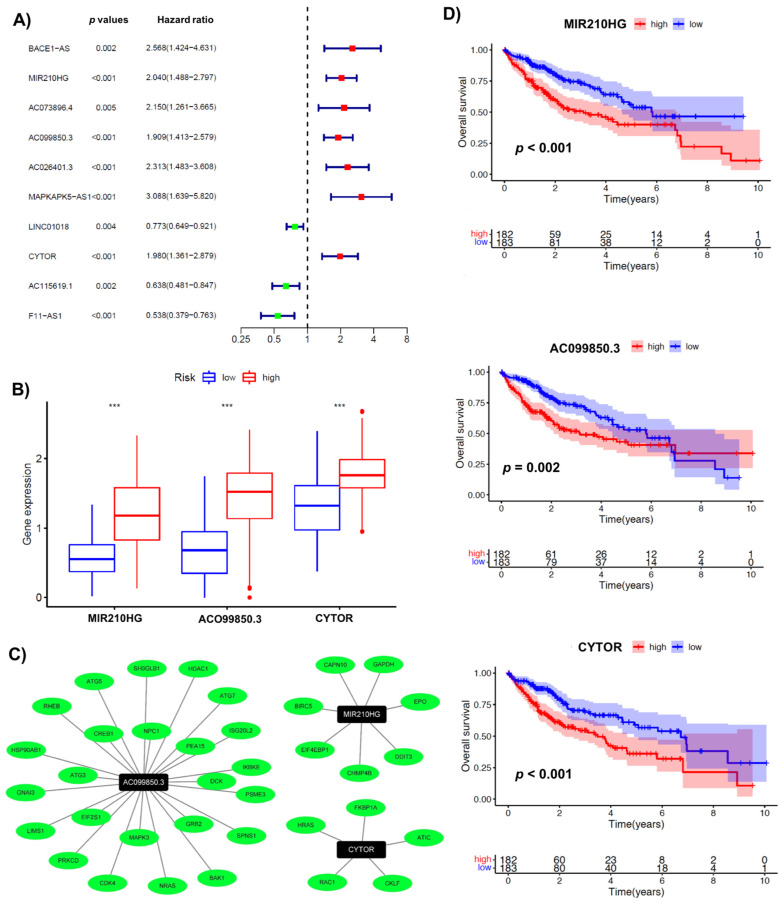
Identification of immuno-autophagy-related lncRNAs with prognostic potential. (**A**) Univariate Cox regression analysis: ten survival-related IARlncRNAs. (**B**) The differential gene expression of IARlncRNAs between high- and low-risk groups. (**C**) A network of prognostic lncRNA (black nodes) with co-expressed genes (green) in HCC. (**D**) Kaplan–Meier survival curves for 3 IARlncRNAs (MIR210HG, AC099850.3, and CYTOR) associated with HCC. *** *p* < 0.001.

The aforementioned 36 immuno-autophagy-related lncRNAs were analyzed in combination with clinical survival data. Univariate Cox regression analysis was performed with a *p*-value of less than 0.01, resulting in the mapping of 10 lncRNAs (BACE1-AS, MIR210HG, AC073896.4, AC099850.3, AC026401.3, MAPKAPK5-AS1, LINC01018, CYTOR, AC115619.1, and F11-AS1) (Figure 1A). In addition, we used a multivariate Cox regression analysis based on the lowest Akaike information criterion (AIC) to determine the β-values that were subsequently used to calculate the risk scores. The analysis revealed three immunoautophagy-related lncRNAs (MIR210HG, AC099850.3, and CYTOR) as the strongest candidates with prognostic potential (Table S1). The correlation between the IARlncRNA of the obtained signature and the genes (AR genes and IR genes) is shown in Figure 1C. Of interest, all these genes showed high expression in the high-risk group (Figure 1B), and were considered unfavorable prognostic determinants (Figure 1D).

### 3.3. Validating the Prognostic Potential of Immuno-Autophagy-Related lncRNA Signature in Low- and High-Risk HCC Groups

**Figure 2:**
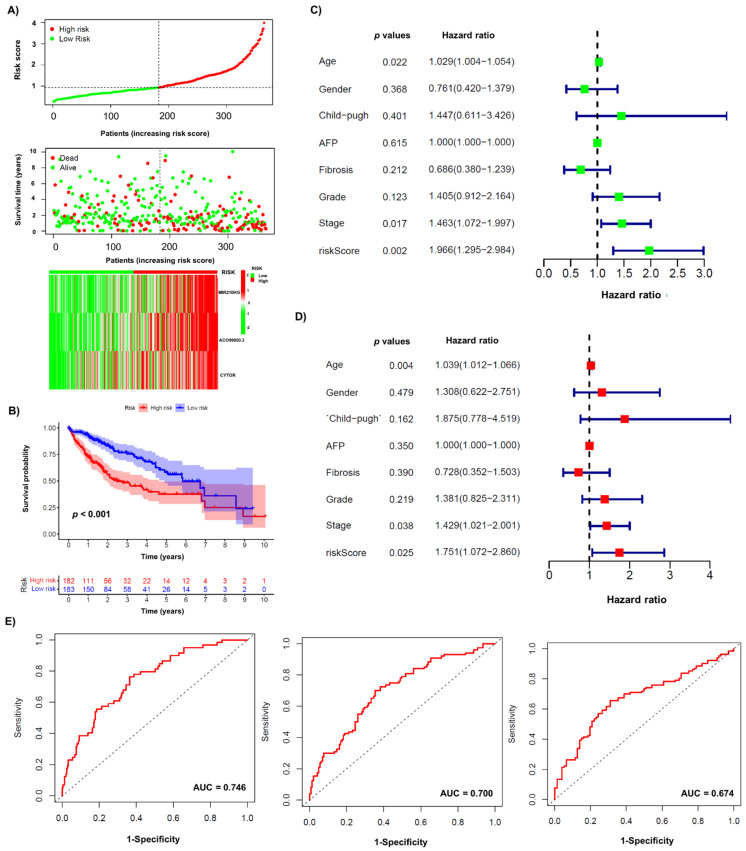
Immunoautophagy-related lncRNA risk score analysis in HCC patients. (**A**) Patient data with low- and high-risk scores (top section), survival status and survival time (middle), and a heatmap of 3 major lncRNAs expressions are shown. (**B**) Kaplan–Meier survival curves for immunoautophagy-related lncRNA risk score for the HCC in TCGA dataset. (**C**) Univariable Cox regression. (**D**) Multivariable Cox regression. (**E**) ROC curve for 1 year (left), 2 years (middle) and 3 years (right).

Next, we determined the functionality of the obtained signature within the low-risk group and high-risk group HCC patients (Figure 2). The scatter plot shows that both survival rates and survival time were lower in the high-risk group compared to the low-risk group (Figure 2A).

Additionally, an expression pattern between lncRNAs and signature risk was observed in the heat map (Figure 2A). The Kaplan–Meier survival curve showed a significant difference in overall survival between the low-risk and high-risk groups (Figure 2B). Notably, the high-risk group showed shorter overall survival compared with the low-risk group. In addition, we performed univariable (Figure 2C) and multivariable Cox (Figure 2D) regression analyses to identify independent prognostic factors with clinical characteristics, and found that age, stage, and risk score were independent predictive determinants of survival in HCC patients. Additionally, an ROC curve was used to confirm the model, for which the AUC values of the risk score for the prediction times of 1, 2, and 3 years were 0.746, 0.700, and 0.674, respectively, for each prediction time (Figure 2E).

### 3.4. Association of Immuno-Autophagy-Related lncRNA Signature with Clinical Characteristics

To determine the association between immuno-autophagy-related lncRNA signature and clinical characteristics, we divided each feature into two groups, such as age (over/under 65 years), gender (male/female), grade (GI–G2/G3–G4), stage (I–II/III–IV), Child–Pugh classification (A/B + C), AFP/alpha-fetoprotein (over/under 400 ng/mL) and fibrosis (with/without) status of patients (Table 1). The analysis showed that a high-risk score was associated significantly with the higher grade (t = 10.918, *p* = 0.001).

### 3.5. Association of Infiltrating Immune Cells and Obtained Signature

**Figure 3:**
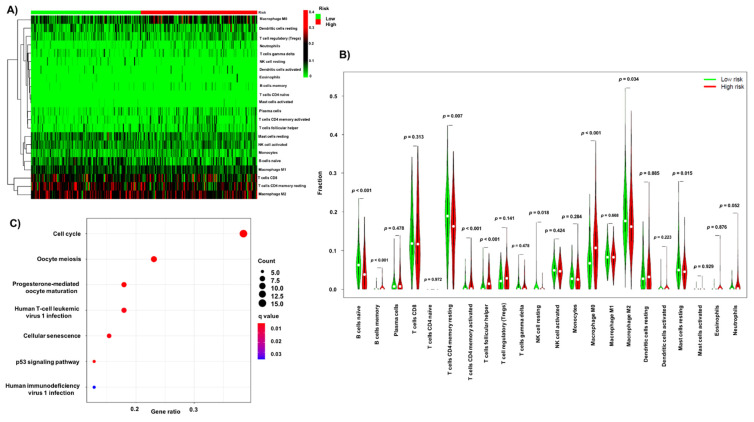
The relationship between immuno-autophagy-related lncRNA signature, infiltration immune cells and potential pathways. (**A**) Heatmap of 22 immune cells in high/low-risk group. (**B**) The fractions of immune cells in high- and low-risk group. (**C**) KEGG analysis.

Considering the obtained signature involved both immune and autophagy determinants, its relationship with immune infiltration cells was investigated. The relative percentages of 22 immune cells in each patient are shown in Figure S2. The distribution of these cells in risk groups is shown in Figure 3A. The Wilcoxon rank sum test was applied to determine the difference between each immune cell in the low- and high-risk groups (Figure 3B). Interestingly, B cells (naïve, *p* < 0.01; memory, *p* < 0.01), T cells CD4 memory (resting, *p* = 0.007; activated, *p* < 0.001), T cells follicular helpers (*p* < 0. 001), NK cells resting (*p* = 0.018), macrophages M0 (*p* < 0.001), macrophages M2 (*p* = 0.034) and mast cells resting (*p* = 0.015) were significantly different between low- and high-risk groups.

### 3.6. GO and KEGG Pathway Enrichment Analysis of the Obtained Signature

We further investigated the cellular and molecular pathways associated with the obtained signature. The differential genes between high- and low-risk groups are listed in Supplementary File S3. The biological/cellular processes obtained from GO analysis (Figure S3) show that the signature is mainly associated with mitosis and chromosome segregation. Additionally, the molecular function of the signature was related to tubulin binding and kinase activity. The KEGG analysis shows that the signature is clearly associated with seven signaling pathways, including cell cycle, oocyte meiosis, progesterone-mediated oocyte maturation, p53 signaling pathway, human T-cell leukemia virus 1 infection, cellular senescence, and human immunodeficiency virus 1 (Figure 3C).

## 4. Discussion

Cancer is a relatively complex disease [6,23], driven primarily by genetic/epigenetic processes that help these cells to proliferate and fuel cancer progression. Overall, the dynamics of dysregulated mechanisms involving several key cellular signaling pathways act as a critical factor for the slow to fast progression of this disease. Among them, autophagy and immune-related processes also play a crucial role in both promoting and suppressing tumor growth. Likewise, the peculiar contribution of long non-coding RNA (IARlncRNA) can also not be excluded. To date, several prognostic signatures involving autophagy-related (AR) and immune-related (IR) genes have been shown [24,25,26], and some have even attempted to combine them with lncRNAs [27,28]. However, to date, no combinatorial signature utilizing IR, AR and lncRNAs has been shown.

With a special focus on hepatocellular carcinoma (HCC), herein, we sought to investigate a possible immuno-autophagy-related long non-coding RNA (IARlncRNA) signature, primarily to predict survival in HCC patients. At first, we selected survival-related IR and AR genes, and combined them lncRNAs to identify ARlncRNAs and IRlncRNAs datasets. Following this, specific sets of ARlncRNAs (*n* = 244) and IRlncRNAs (*n* = 36) were generated, and then a preliminary signature of IARlncRNAs (*n* = 36) was derived from the aforementioned data sets. Among them, 10 IARlncRNAs (BACE1-AS, MIR210HG, AC073896.4, AC099850.3, AC026401.3, MAPKAPK5-AS1, LINC01018, CYTOR, AC115619.1, and F11-AS1) were found to be associated with survival. Of importance, three of them (MIR210HG, AC099850.3, and CYTOR) displayed a robust prognostic signature with unfavorable prognosis. Previously, these three IARlncRNAs had all been implicated in HCC; for instance, it has been shown that the silencing of MIR210HG expression leads to the inhibition of HCC tumor growth [29]. Similarly, CYTOR has been shown to promote HCC proliferation, and its disruption inhibited HCC growth [30,31]. Additionally, AC099850.3 has been shown to increase proliferation and migration in HCC [32], thus providing strong evidence for the utility of our prognostic signature in the clinical spectrum of HCC patients.

We next determined the functionality of the obtained signature within the HCC patient groups, and found that both survival rates and survival time were significantly low in the high-risk group. In addition, an inverse expression pattern was observed in lncRNAs and risk groups. Of interest, among several clinical characteristics, risk score was found to be an independent predictive determinant of survival in HCC patients. We also examined the relationship between the obtained signature and the infiltrating immune cells. The analysis showed that a higher proportion of naïve B cells, resting memory CD4 T cells, resting NK cells, M2 macrophages, and resting mast cells predominated in the low-risk group, whereas the proportion of memory B cells, activated memory CD4 T cells, follicular helper T cells, and M0 macrophages was specific for the high-risk group. GO analysis showed that differential gene expressions between risk groups were significantly enriched in biological processes (mitosis and chromosome segregation), cellular components (chromosomes), and molecular functions (tubulin binding and kinase activity). In addition, seven defined signaling pathways (cell cycle, oocyte meiosis, progesterone-mediated oocyte maturation, p53 signaling pathway, human T-cell leukemia virus 1 infection, cellular senescence, and human immunodeficiency virus 1) were found to be associated with the obtained signature. To our knowledge, we have presented for the first time an immuno-autophagy-related long non-coding RNA (IARlncRNA) signature prognostic ability in hepatocellular carcinoma. It is worth mentioning that molecular validation of this obtained signature using clinical samples is required. Prognostic models for HCC based on lncRNAs have also been reported previously. For instance, a recent study identified five autophagy-related long non-coding RNAs (AR-lncRNAs) (including TMCC1-AS1, PLBD1-AS1, MKLN1-AS, LINC01063, and CYTOR) for HCC patients from the TCGA database [27]. Likewise, one independent study described four-immune-related-LncRNA signatures for predicting the prognosis and guiding the application of immunotherapy in HCC [33]. However, it is worth mentioning that the heterogeneity within clinical samples submitted to repositories (as previously described by Sharma et al. [34]) and especially the selection of different computational analytical methods makes these predictive markers less effective in the clinical environment. In the present study, we have provided a detailed description of the methods used in our analysis, which offers a platform for methodological compression to enable similar analyses in HCC or in other cancers.

## 5. Conclusions

The immuno-autophagy-related long non-coding RNA (IARlncRNA) signature we established exhibits a prognostic ability in hepatocellular carcinoma.

## Figures and Tables

**Table 1:** The relation between risk of signature with clinical features.

	Risk	Total	High Risk	Low Risk	t	*p* Value
Age	<65	95 (58.28%)	46 (63.89%)	49 (53.85%)	1.280	0.258
	≥65	68 (41.72%)	26 (36.11%)	42 (46.15%)		
Gender	Female	50 (30.67%)	24 (33.33%)	26 (28.57%)	0.234	0.629
	Male	113 (69.33%)	48 (66.67%)	65 (71.43%)		
Child–Pugh	A	147 (90.18%)	64 (88.89%)	83 (91.21%)	0.053	0.819
	B + C	16 (9.82%)	8 (11.11%)	8 (8.79%)		
AFP	≥400	30 (18.4%)	17 (23.61%)	13 (14.29%)	1.748	0.186
	<400	133 (81.6%)	55 (76.39%)	78 (85.71%)		
Fibrosis	Fibrosis	113 (69.33%)	50 (69.44%)	63 (69.23%)	0	1
	No Fibrosis	50 (30.67%)	22 (30.56%)	28 (30.77%)		
Grade	G1–G2	99 (60.74%)	33 (45.83%)	66 (72.53%)	10.918	0.001 **
	G3–G4	64 (39.26%)	39 (54.17%)	25 (27.47%)		
Stage	Stage I–II	131 (80.37%)	57 (79.17%)	74 (81.32%)	0.021	0.885
	Stage III–IV	32 (19.63%)	15 (20.83%)	17 (18.68%)		

** *p* < 0.01.

## Data Availability

The data set in this study can be found at https://xenabrowser.net/datapages/ as accessed on 22 October 2021. The TCGA-LIHC dataset is GDC TCGA Liver Cancer (LIHC) (14 datasets).

## Supplementary Materials

The following are available online at https://www.mdpi.com/article/10.3390/biology10121301/s1, Supplementary File S1, Supplementary File S2, Supplementary File S3, Table S1: Information of multivariable Cox regression, Figure S1: Flow chart illustrating the defined strategy in the study, Figure S2: Percentage of infiltration immune cells in patients, Figure S3: GO analysis.
