# Supplementary material for: Immunoautophagy-Related Long Noncoding RNA (IAR-lncRNA) Signature Predicts Survival in Hepatocellular Carcinoma
Source: Biology (Basel). 2021 Dec 9;10(12):1301. doi: 10.3390/biology10121301 (PMC8698564; doi:10.3390/biology10121301)

Supplementary Files:

Supplementary Table S1: Information of multivariable Cox regression

LncRNA	β	Hazard ratio
MIR210HG	0.359	1.432
AC099850.3	0.555	1.741
CYTOR	0.604	1.830

Supplementary Figure S1: Flow chart illustrating the defined strategy in the study

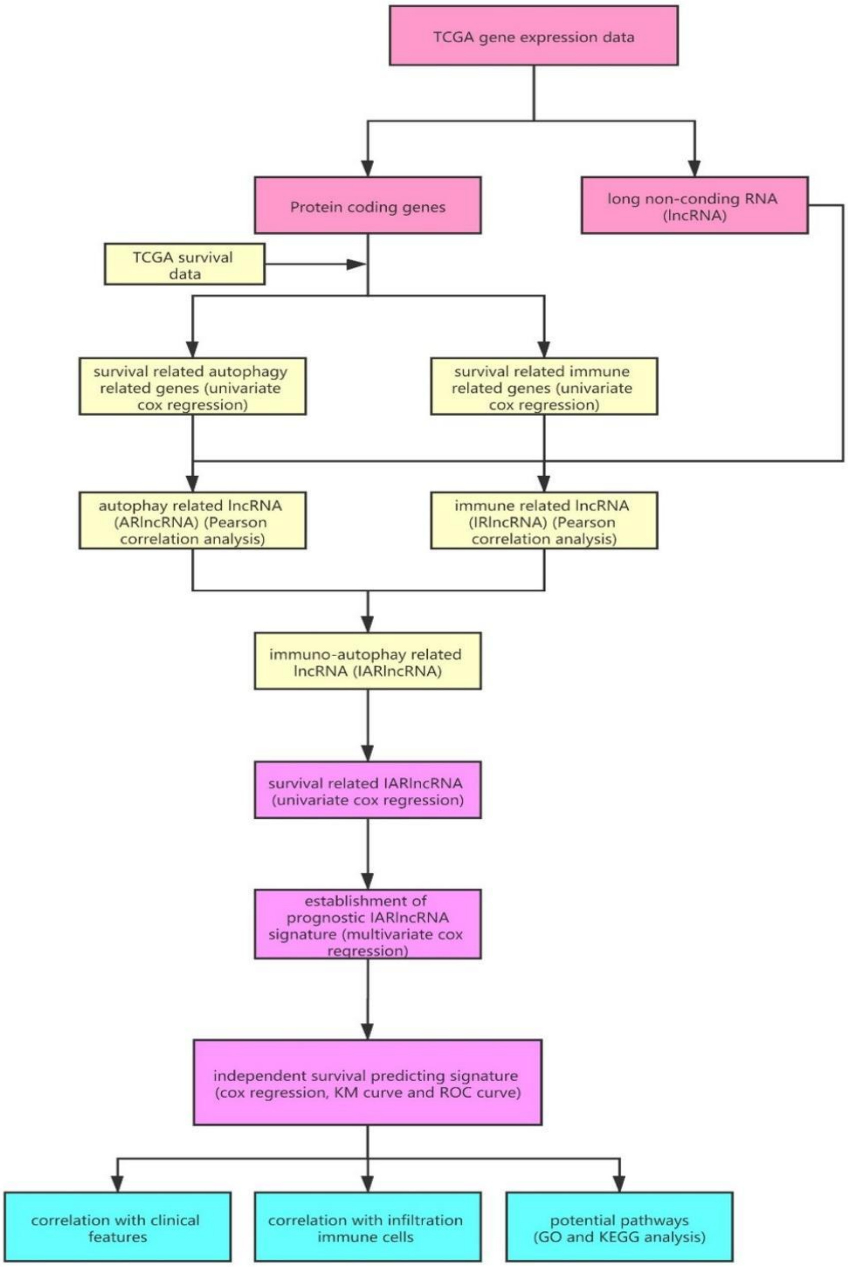

Supplementary Figure S2: Percentage of infiltration immune cells in patients

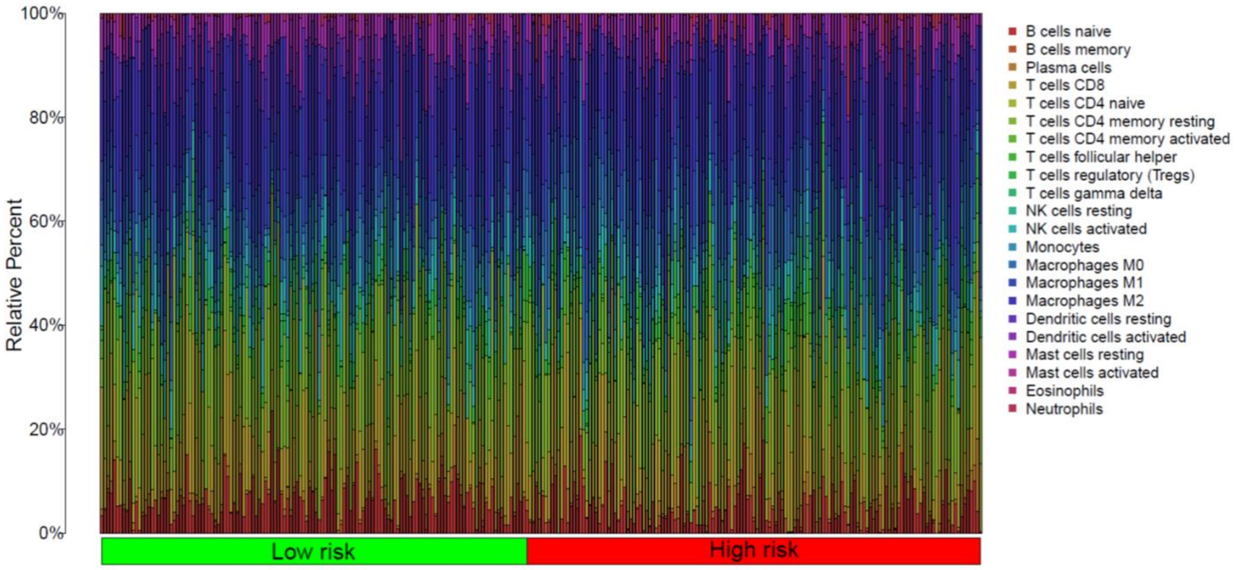

Supplementary Figure S3: GO analysis

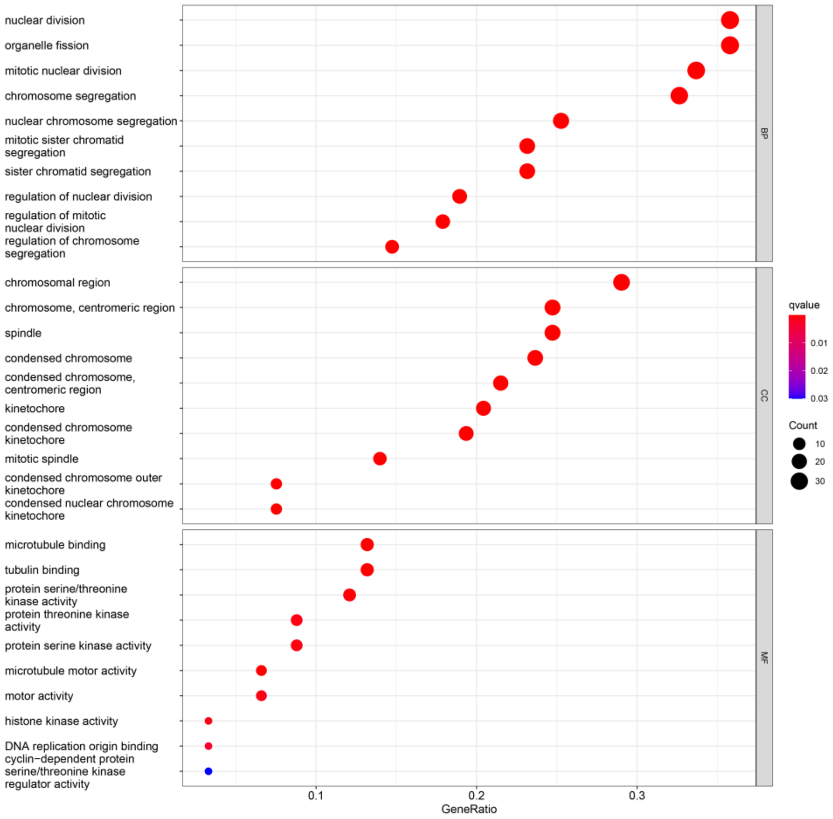

Supplement: Supplementary file 1 [file biology-10-01301-s001.zip › biology-1472829-supplementary.pdf]
