# Supplementary material for: Immunoautophagy-Related Long Noncoding RNA (IAR-lncRNA) Signature Predicts Survival in Hepatocellular Carcinoma
Source: Biology (Basel). 2021 Dec 9;10(12):1301. doi: 10.3390/biology10121301 (PMC8698564; doi:10.3390/biology10121301)
Supplement: Supplementary file 1 [file biology-10-01301-s001.zip › Supplementary File S1.pdf]

ARGene	lncRNA	cor	pvalue	relation
ATG5	AL133338.1	0.422288473	3.23E-17	postive
PRKCD	FOXD2-AS1	0.445677785	3.26E-19	postive
IKBKE	FOXD2-AS1	0.412022176	2.17E-16	postive
GABARAPL1	FOXD2-AS1	-0.461309498	1.24E-20	negative
EIF4EBP1	FOXD2-AS1	0.416901368	8.84E-17	postive
CHMP4B	FOXD2-AS1	0.453473236	6.52E-20	postive
CDKN2A	FOXD2-AS1	0.486848923	4.04E-23	postive
CAPN10	FOXD2-AS1	0.493592446	8.22E-24	postive
BIRC5	FOXD2-AS1	0.55224004	1.59E-30	postive
IKBKE	PRKAG2-AS1	-0.437624047	1.65E-18	negative
BAK1	AL031722.1	-0.44958911	1.46E-19	negative
ATIC	AL031722.1	-0.414338966	1.42E-16	negative
CDKN2A	AC008771.1	0.410978646	2.62E-16	postive
BIRC5	AC008771.1	0.479288476	2.31E-22	postive
RAC1	SNHG6	0.449012902	1.65E-19	postive
IKBKE	SNHG6	0.404969789	7.74E-16	postive
FKBP1A	SNHG6	0.543463848	1.95E-29	postive
CHMP4B	SNHG6	0.427614006	1.17E-17	postive
BIRC5	SNHG6	0.44545299	3.42E-19	postive
ATIC	SNHG6	0.551197248	2.15E-30	postive
ATIC	CASC19	0.423136264	2.75E-17	postive
RAC1	AC015912.3	0.412581968	1.96E-16	postive
GAPDH	AC015912.3	0.415214822	1.21E-16	postive
FKBP1A	AC015912.3	0.443937548	4.65E-19	postive
EIF4EBP1	AC015912.3	0.414738637	1.32E-16	postive
CHMP4B	AC015912.3	0.431849719	5.15E-18	postive
BIRC5	AC015912.3	0.408290072	4.27E-16	postive
ATIC	AC015912.3	0.402999753	1.10E-15	postive
MAPK3	AL355574.1	0.431330536	5.70E-18	postive
CAPN10	AL355574.1	0.461311101	1.24E-20	postive
ATIC	AL355574.1	0.423109231	2.76E-17	postive
IKBKE	AC008549.1	-0.477252697	3.67E-22	negative
CHMP4B	AC008549.1	-0.438436712	1.41E-18	negative
BAK1	AC008549.1	-0.500799341	1.44E-24	negative
RAC1	AC023043.3	0.423212937	2.71E-17	postive
EIF4EBP1	AC023043.3	0.436289198	2.15E-18	postive
DDIT3	AC023043.3	0.489967644	1.94E-23	postive
CHMP4B	AC023043.3	0.482284736	1.16E-22	postive
CAPN10	AC023043.3	0.453502286	6.48E-20	postive
BIRC5	AC023043.3	0.534596664	2.29E-28	postive
BAK1	AC023043.3	0.408508703	4.10E-16	postive
RAC1	AC009686.2	0.450151582	1.30E-19	postive
ATIC	AC009686.2	0.422408393	3.15E-17	postive
SPNS1	WAC-AS1	0.403834999	9.47E-16	postive
PEA15	WAC-AS1	0.462286636	1.00E-20	postive
NPC1	WAC-AS1	0.460012686	1.63E-20	postive

MAPK3	WAC-AS1	0.404463009	8.47E-16	postive
GNAI3	WAC-AS1	0.434831578	2.87E-18	postive
CHMP4B	WAC-AS1	0.430470772	6.73E-18	postive
CAPN10	WAC-AS1	0.424443594	2.14E-17	postive
BAK1	WAC-AS1	0.412953224	1.83E-16	postive
ATG9A	WAC-AS1	0.422480268	3.11E-17	postive
ATG4B	WAC-AS1	0.439396903	1.16E-18	postive
SPNS1	AC068580.1	0.568822632	1.12E-32	postive
SH3GLB1	AC068580.1	0.424562205	2.10E-17	postive
NPC1	AC068580.1	0.532554146	4.00E-28	postive
RAC1	AC018645.3	0.510779587	1.20E-25	postive
RAB24	AC092535.5	0.436098923	2.24E-18	postive
CAPN10	AC092535.5	0.519140718	1.41E-26	postive
ATG4B	AC092535.5	0.432322898	4.70E-18	postive
SPNS1	MID1IP1-AS1	0.490901714	1.56E-23	postive
NPC1	MID1IP1-AS1	0.44708305	2.45E-19	postive
SPNS1	ASB16-AS1	0.468020261	2.88E-21	postive
MAPK3	ASB16-AS1	0.422878963	2.88E-17	postive
BIRC5	ASB16-AS1	0.410455135	2.89E-16	postive
PRKCD	LINC02041	0.411244319	2.50E-16	postive
CHMP4B	LINC02041	0.422343333	3.19E-17	postive
RAC1	MAFG-DT	0.422024266	3.39E-17	postive
MAPK3	MAFG-DT	0.482139012	1.20E-22	postive
GAPDH	MAFG-DT	0.406608325	5.78E-16	postive
FKBP1A	MAFG-DT	0.411285058	2.48E-16	postive
CHMP4B	MAFG-DT	0.47228017	1.12E-21	postive
BIRC5	MAFG-DT	0.529115726	1.01E-27	postive
ATIC	MAFG-DT	0.452682376	7.69E-20	postive
FKBP1A	AL360181.1	-0.425940479	1.61E-17	negative
HDAC1	AL050341.2	0.441356943	7.84E-19	postive
GNAI3	AL050341.2	0.465180642	5.35E-21	postive
CAPN10	AL050341.2	0.407700476	4.75E-16	postive
BIRC5	AL050341.2	0.405755681	6.73E-16	postive
IKBKE	AP001783.1	-0.450771908	1.14E-19	negative
HDAC1	AP001783.1	-0.44354411	5.04E-19	negative
BAK1	AP001783.1	-0.401344004	1.47E-15	negative
ATIC	AP001783.1	-0.444406068	4.23E-19	negative
RAC1	AL035461.2	0.475284215	5.73E-22	postive
PRKCD	AL035461.2	0.458154198	2.43E-20	postive
MTMR14	AL035461.2	0.419158087	5.81E-17	postive
IKBKE	AL035461.2	0.405617079	6.90E-16	postive
HDAC1	AL035461.2	0.401010588	1.56E-15	postive
GABARAPL1	AL035461.2	-0.430650842	6.50E-18	negative
FKBP1A	AL035461.2	0.588057025	2.51E-35	postive
EIF4EBP1	AL035461.2	0.501189351	1.31E-24	postive
CHMP4B	AL035461.2	0.59501892	2.49E-36	postive
CDKN2A	AL035461.2	0.427752621	1.14E-17	postive

CAPN10	AL035461.2	0.558627126	2.44E-31	postive
BIRC5	AL035461.2	0.669770746	7.44E-49	postive
ATIC	AL035461.2	0.412142333	2.12E-16	postive
ATG4B	AL035461.2	0.574577805	1.89E-33	postive
PEA15	LINC01093	-0.41314694	1.77E-16	negative
HSP90AB1	LINC01093	-0.433964168	3.41E-18	negative
GAPDH	LINC01093	-0.418114859	7.06E-17	negative
ATIC	LINC01093	-0.528656757	1.14E-27	negative
GAPDH	AL445524.1	0.422374511	3.17E-17	postive
CDKN2A	AC106820.3	0.452484074	8.01E-20	postive
BIRC5	AC106820.3	0.462630716	9.30E-21	postive
PRKCD	LINC02381	0.411551988	2.36E-16	postive
IKBKE	LINC02381	0.418187594	6.96E-17	postive
FKBP1A	LINC02381	0.421654311	3.64E-17	postive
CHMP4B	LINC02381	0.400812675	1.62E-15	postive
CAPN10	LINC02381	0.429525765	8.09E-18	postive
ATG4B	LINC02381	0.428890162	9.14E-18	postive
RAC1	AC026740.1	0.420540574	4.48E-17	postive
IKBKE	AC026740.1	0.440311375	9.67E-19	postive
CAPN10	AC026740.1	0.439422352	1.15E-18	postive
GNAI3	Z95115.1	0.406948346	5.43E-16	postive
BIRC5	Z95115.1	0.422035526	3.38E-17	postive
RAC1	AP001505.1	0.463034194	8.53E-21	postive
FKBP1A	AP001505.1	0.464969919	5.61E-21	postive
EIF4EBP1	AP001505.1	0.46370502	7.38E-21	postive
CHMP4B	AP001505.1	0.482345503	1.15E-22	postive
CAPN10	AP001505.1	0.465320394	5.19E-21	postive
BIRC5	AP001505.1	0.532036523	4.60E-28	postive
ATIC	AP001505.1	0.414257313	1.44E-16	postive
ATG4B	AP001505.1	0.523480667	4.53E-27	postive
RAB24	MELTF-AS1	0.413265347	1.73E-16	postive
PRKCD	MELTF-AS1	0.48279181	1.04E-22	postive
MAPK3	MELTF-AS1	0.42428325	2.21E-17	postive
IKBKE	MELTF-AS1	0.469299404	2.17E-21	postive
HDAC1	MELTF-AS1	0.430559303	6.62E-18	postive
FKBP1A	MELTF-AS1	0.407228869	5.17E-16	postive
CHMP4B	MELTF-AS1	0.536040927	1.54E-28	postive
CAPN10	MELTF-AS1	0.556496596	4.57E-31	postive
BIRC5	MELTF-AS1	0.575060569	1.62E-33	postive
BAK1	MELTF-AS1	0.469735688	1.97E-21	postive
ATG4B	MELTF-AS1	0.546912683	7.35E-30	postive
PEA15	DCXR-DT	-0.412888395	1.85E-16	negative
IKBKE	DCXR-DT	-0.511815604	9.25E-26	negative
BAK1	DCXR-DT	-0.477807519	3.24E-22	negative
SPNS1	AC080112.1	0.434933529	2.81E-18	postive
RAC1	SNHG17	0.4401543	9.97E-19	postive
RAB24	SNHG17	0.455093837	4.64E-20	postive

FKBP1A	SNHG17	0.537671285	9.83E-29	postive
DDIT3	SNHG17	0.429068918	8.83E-18	postive
CHMP4B	SNHG17	0.52612122	2.25E-27	postive
CAPN10	SNHG17	0.508904999	1.93E-25	postive
BIRC5	SNHG17	0.401745838	1.37E-15	postive
BAK1	SNHG17	0.403872375	9.41E-16	postive
ATIC	SNHG17	0.551660322	1.88E-30	postive
ATG4B	SNHG17	0.51557225	3.55E-26	postive
SPNS1	RAB11B-AS1	-0.459137293	1.97E-20	negative
SH3GLB1	RAB11B-AS1	-0.419831224	5.12E-17	negative
PEA15	RAB11B-AS1	-0.413125619	1.77E-16	negative
NPC1	RAB11B-AS1	-0.479065693	2.43E-22	negative
ATG3	RAB11B-AS1	-0.410067042	3.10E-16	negative
IKBKE	AC012313.9	-0.478754553	2.61E-22	negative
CHMP4B	AC012313.9	-0.400844761	1.61E-15	negative
BAK1	AC012313.9	-0.457718385	2.66E-20	negative
ATIC	AC012313.9	-0.400773688	1.63E-15	negative
IKBKE	LINC02754	-0.487663894	3.34E-23	negative
BAK1	LINC02754	-0.42216995	3.30E-17	negative
SH3GLB1	ZSCAN16-AS1	-0.456658752	3.33E-20	negative
CAPN10	LINC00685	0.452933621	7.30E-20	postive
ATG4B	LINC00685	0.507335892	2.86E-25	postive
CDKN2A	AL441992.1	0.448969702	1.66E-19	postive
PRKCD	TMPO-AS1	0.407081335	5.31E-16	postive
CDKN2A	TMPO-AS1	0.429033891	8.89E-18	postive
BIRC5	TMPO-AS1	0.64375773	4.24E-44	postive
IKBKE	LINC00623	0.444219569	4.39E-19	postive
FKBP1A	LINC00623	0.454691216	5.05E-20	postive
CHMP4B	LINC00623	0.418095182	7.08E-17	postive
FKBP1A	EP300-AS1	-0.432483982	4.55E-18	negative
RAC1	GAS5	0.417395788	8.07E-17	postive
RAB24	GAS5	0.49629812	4.29E-24	postive
HSP90AB1	GAS5	0.412932531	1.84E-16	postive
FKBP1A	GAS5	0.452811395	7.48E-20	postive
CAPN10	GAS5	0.450474556	1.22E-19	postive
ATIC	GAS5	0.622613781	1.47E-40	postive
ATG4B	GAS5	0.428618513	9.64E-18	postive
CHMP4B	LINC01836	0.435250443	2.64E-18	postive
RAC1	AC138207.5	0.455313753	4.43E-20	postive
MAPK3	AC138207.5	0.41217818	2.11E-16	postive
IKBKE	AC138207.5	0.537392662	1.06E-28	postive
FKBP1A	AC138207.5	0.524067999	3.88E-27	postive
CHMP4B	AC138207.5	0.46954791	2.06E-21	postive
BAK1	AC138207.5	0.417641466	7.71E-17	postive
ATG7	AC138207.5	0.477276537	3.65E-22	postive
RAC1	MIR4435-2HG	0.569713618	8.55E-33	postive
MAPK3	MIR4435-2HG	0.44696468	2.51E-19	postive

IKBKE	MIR4435-2HG	0.512259875	8.27E-26	postive
GAPDH	MIR4435-2HG	0.457029873	3.08E-20	postive
FKBP1A	MIR4435-2HG	0.605509329	6.87E-38	postive
CHMP4B	MIR4435-2HG	0.572765933	3.32E-33	postive
CAPN10	MIR4435-2HG	0.459379289	1.87E-20	postive
BAK1	MIR4435-2HG	0.477262717	3.66E-22	postive
ATIC	MIR4435-2HG	0.523271215	4.79E-27	postive
ATG7	MIR4435-2HG	0.412525981	1.98E-16	postive
CAPN10	THAP9-AS1	0.409976877	3.15E-16	postive
FKBP1A	LINC02037	-0.464851622	5.75E-21	negative
RAB24	LMNTD2-AS1	0.416646987	9.27E-17	postive
CAPN10	LMNTD2-AS1	0.472440821	1.08E-21	postive
ATG4B	LMNTD2-AS1	0.432144752	4.86E-18	postive
IKBKE	PCED1B-AS1	0.560396787	1.44E-31	postive
FKBP1A	PCED1B-AS1	0.485380497	5.69E-23	postive
ATG7	PCED1B-AS1	0.423484993	2.57E-17	postive
HSP90AB1	LINC02428	-0.410384742	2.92E-16	negative
SPNS1	AC005288.1	0.693386413	1.30E-53	postive
SH3GLB1	AC005288.1	0.622176189	1.72E-40	postive
RHEB	AC005288.1	0.427139059	1.28E-17	postive
NPC1	AC005288.1	0.595729509	1.96E-36	postive
GNAI3	AC005288.1	0.60710067	3.94E-38	postive
ATG3	AC005288.1	0.475430246	5.54E-22	postive
NPC1	AC005332.6	0.439168744	1.21E-18	postive
ATG4B	BAIAP2-DT	0.402494592	1.20E-15	postive
EIF4EBP1	AC093673.1	0.443288032	5.31E-19	postive
CHMP4B	AC093673.1	0.474526193	6.79E-22	postive
BIRC5	AC093673.1	0.401528852	1.42E-15	postive
SH3GLB1	NORAD	0.45662417	3.36E-20	postive
NPC1	NORAD	0.410350052	2.94E-16	postive
GNAI3	NORAD	0.45749809	2.79E-20	postive
SPNS1	AL450326.1	0.465184973	5.35E-21	postive
RAB24	ARRDC1-AS1	0.418288117	6.83E-17	postive
CDKN2A	ARRDC1-AS1	0.442875744	5.77E-19	postive
CAPN10	ARRDC1-AS1	0.465717392	4.76E-21	postive
BIRC5	ARRDC1-AS1	0.423759515	2.44E-17	postive
ATG4B	ARRDC1-AS1	0.455280658	4.46E-20	postive
RAC1	SNHG7	0.414164179	1.47E-16	postive
RAB24	SNHG7	0.544956366	1.28E-29	postive
PRKCD	SNHG7	0.439044859	1.25E-18	postive
FKBP1A	SNHG7	0.473968582	7.69E-22	postive
CHMP4B	SNHG7	0.46598003	4.50E-21	postive
CAPN10	SNHG7	0.548064035	5.29E-30	postive
BIRC5	SNHG7	0.479168302	2.38E-22	postive
ATIC	SNHG7	0.526342838	2.12E-27	postive
ATG4B	SNHG7	0.548984666	4.06E-30	postive
RAB24	BACE1-AS	0.509938663	1.49E-25	postive

PRKCD	BACE1-AS	0.480355755	1.81E-22	postive
PEA15	BACE1-AS	0.423206788	2.71E-17	postive
NPC1	BACE1-AS	0.409577064	3.38E-16	postive
IKBKE	BACE1-AS	0.462206069	1.02E-20	postive
HDAC1	BACE1-AS	0.455564281	4.20E-20	postive
CHMP4B	BACE1-AS	0.408974227	3.77E-16	postive
CAPN10	BACE1-AS	0.629742744	1.01E-41	postive
BIRC5	BACE1-AS	0.447077662	2.45E-19	postive
BAK1	BACE1-AS	0.422464822	3.12E-17	postive
ATIC	BACE1-AS	0.46778208	3.03E-21	postive
ATG9A	BACE1-AS	0.449337496	1.54E-19	postive
ATG4B	BACE1-AS	0.576966707	8.91E-34	postive
SH3GLB1	AL355488.1	0.424475332	2.13E-17	postive
RAB24	AL355488.1	0.466137984	4.35E-21	postive
PRKCD	AL355488.1	0.458706319	2.16E-20	postive
NPC1	AL355488.1	0.457974694	2.52E-20	postive
IKBKE	AL355488.1	0.417580215	7.79E-17	postive
HDAC1	AL355488.1	0.414356457	1.41E-16	postive
GNAI3	AL355488.1	0.49497366	5.90E-24	postive
CAPN10	AL355488.1	0.603645738	1.31E-37	postive
BIRC5	AL355488.1	0.491111244	1.48E-23	postive
ATG4B	AL355488.1	0.527451618	1.58E-27	postive
IKBKE	AC133552.5	-0.527316801	1.64E-27	negative
SPNS1	AC068580.3	0.497141513	3.50E-24	postive
NPC1	AC068580.3	0.467724775	3.07E-21	postive
RAB24	PTOV1-AS2	0.458604122	2.21E-20	postive
PRKCD	PTOV1-AS2	0.436023276	2.27E-18	postive
NPC1	PTOV1-AS2	0.465224241	5.30E-21	postive
HDAC1	PTOV1-AS2	0.438894672	1.28E-18	postive
CAPN10	PTOV1-AS2	0.607090676	3.95E-38	postive
ATG4B	PTOV1-AS2	0.529610449	8.86E-28	postive
CAPN10	PSMA3-AS1	0.409378172	3.51E-16	postive
ATG4B	PSMA3-AS1	0.446072215	3.01E-19	postive
BIRC5	AL031673.1	0.400070765	1.84E-15	postive
RAC1	PRKAR1B-AS1	0.417798132	7.48E-17	postive
IKBKE	PRKAR1B-AS1	0.45654609	3.41E-20	postive
FKBP1A	PRKAR1B-AS1	0.50319918	7.98E-25	postive
CHMP4B	PRKAR1B-AS1	0.447861564	2.09E-19	postive
IKBKE	HNF4A-AS1	-0.461716856	1.13E-20	negative
HDAC1	HNF4A-AS1	-0.409487116	3.44E-16	negative
BAK1	HNF4A-AS1	-0.459323417	1.89E-20	negative
SPNS1	FGD5-AS1	0.587192266	3.34E-35	postive
SH3GLB1	FGD5-AS1	0.568570735	1.21E-32	postive
RHEB	FGD5-AS1	0.415920315	1.06E-16	postive
PRKCD	FGD5-AS1	0.400133201	1.82E-15	postive
NPC1	FGD5-AS1	0.561827517	9.39E-32	postive
GNAI3	FGD5-AS1	0.566456913	2.32E-32	postive

ATG3	FGD5-AS1	0.427135398	1.28E-17	postive
IKBKE	AC009005.1	0.426772136	1.37E-17	postive
GABARAPL1	AC009005.1	-0.406230264	6.18E-16	negative
CAPN10	AC009005.1	0.426818893	1.36E-17	postive
BIRC5	AC009005.1	0.520870719	8.99E-27	postive
BAK1	AC009005.1	0.406005054	6.43E-16	postive
ATIC	AC009005.1	0.418616321	6.43E-17	postive
NPC1	AC008735.2	0.50387286	6.76E-25	postive
CAPN10	AC008735.2	0.497956765	2.87E-24	postive
ATG4B	AC008735.2	0.439555605	1.12E-18	postive
PEA15	LINC01348	-0.416229901	1.00E-16	negative
PEA15	AC010336.2	-0.43199471	5.01E-18	negative
IKBKE	AC010336.2	-0.585006279	6.80E-35	negative
HDAC1	AC010336.2	-0.420450583	4.56E-17	negative
CAPN10	AC010336.2	-0.404313563	8.70E-16	negative
BAK1	AC010336.2	-0.493404143	8.59E-24	negative
ATIC	AC010336.2	-0.423410869	2.61E-17	negative
IKBKE	AC012640.2	-0.420998439	4.11E-17	negative
BIRC5	SNHG25	0.442092745	6.76E-19	postive
ATG4B	SNHG25	0.439230544	1.20E-18	postive
SPNS1	ANKRD10-IT1	0.456602317	3.37E-20	postive
SH3GLB1	ANKRD10-IT1	0.465081377	5.47E-21	postive
NPC1	ANKRD10-IT1	0.468134978	2.81E-21	postive
GNAI3	ANKRD10-IT1	0.421446943	3.78E-17	postive
RAB24	ASMTL-AS1	0.441543632	7.55E-19	postive
RAC1	AGAP2-AS1	0.419101908	5.87E-17	postive
FKBP1A	AGAP2-AS1	0.44266167	6.02E-19	postive
CHMP4B	AGAP2-AS1	0.466833577	3.73E-21	postive
RAC1	AC011468.1	0.448525113	1.82E-19	postive
FKBP1A	AC011468.1	0.436734818	1.97E-18	postive
CHMP4B	AC011468.1	0.476933028	3.95E-22	postive
CAPN10	AC011468.1	0.488697008	2.62E-23	postive
ATG4B	AC011468.1	0.550461605	2.66E-30	postive
FKBP1A	AC105105.2	-0.403452032	1.01E-15	negative
PEA15	AC068338.2	-0.419181556	5.78E-17	negative
IKBKE	AC068338.2	-0.493494945	8.41E-24	negative
BAK1	AC068338.2	-0.417686761	7.64E-17	negative
SPNS1	AC016888.1	0.703879486	7.09E-56	postive
NPC1	AC016888.1	0.473970047	7.69E-22	postive
GNAI3	AC016888.1	0.472668483	1.03E-21	postive
SPNS1	LINC00106	0.401259128	1.49E-15	postive
RAC1	GIHCG	0.422088779	3.35E-17	postive
MTMR14	GIHCG	0.482327415	1.15E-22	postive
MAPK3	GIHCG	0.424250142	2.22E-17	postive
FKBP1A	GIHCG	0.538134617	8.65E-29	postive
CHMP4B	GIHCG	0.489176221	2.34E-23	postive
CDKN2A	GIHCG	0.469299005	2.17E-21	postive

BIRC5	GIHCG	0.526111796	2.26E-27	postive
ATIC	GIHCG	0.444457358	4.19E-19	postive
ATG4B	GIHCG	0.427928568	1.10E-17	postive
BIRC5	PIK3CD-AS2	0.429142709	8.71E-18	postive
ATIC	PIK3CD-AS2	0.414878349	1.29E-16	postive
PRKCD	AC004862.1	-0.402752275	1.15E-15	negative
IKBKE	AC004862.1	-0.447117949	2.43E-19	negative
BAK1	AC004862.1	-0.412172336	2.11E-16	negative
SPNS1	EBLN3P	0.4374889	1.70E-18	postive
SH3GLB1	EBLN3P	0.551609447	1.91E-30	postive
NPC1	EBLN3P	0.412862575	1.86E-16	postive
GNAI3	EBLN3P	0.47928305	2.32E-22	postive
BIRC5	CEBPA-DT	0.410923424	2.65E-16	postive
IKBKE	AL049840.5	0.417496941	7.92E-17	postive
FKBP1A	AL049840.5	0.489696514	2.07E-23	postive
CAPN10	AL049840.5	0.538330522	8.19E-29	postive
ATIC	AL049840.5	0.458269225	2.37E-20	postive
ATG4B	AL049840.5	0.54596432	9.62E-30	postive
PRKCD	AC239800.2	-0.477667074	3.34E-22	negative
NPC1	AC239800.2	-0.420960516	4.14E-17	negative
IKBKE	AC239800.2	-0.400542668	1.69E-15	negative
IKBKE	AL355102.4	0.50672501	3.33E-25	postive
CHMP4B	AL355102.4	0.405690008	6.81E-16	postive
CAPN10	AL355102.4	0.44030884	9.67E-19	postive
BAK1	AL355102.4	0.445580093	3.33E-19	postive
SPNS1	AC012065.2	-0.438814148	1.30E-18	negative
SPNS1	AC005332.5	0.417996557	7.21E-17	postive
RHEB	AC005332.5	0.412926484	1.84E-16	postive
RAB24	AC005332.5	0.515095372	4.01E-26	postive
NPC1	AC005332.5	0.501520007	1.21E-24	postive
CAPN10	AC005332.5	0.516169324	3.04E-26	postive
BIRC5	AC005332.5	0.508331188	2.23E-25	postive
BAK1	AC005332.5	0.406153591	6.27E-16	postive
ATG9A	AC005332.5	0.401526941	1.42E-15	postive
ATG4B	AC005332.5	0.418555802	6.50E-17	postive
GAPDH	MIR210HG	0.405550101	6.98E-16	postive
EIF4EBP1	MIR210HG	0.41169558	2.30E-16	postive
DDIT3	MIR210HG	0.420418022	4.59E-17	postive
CHMP4B	MIR210HG	0.428466171	9.92E-18	postive
CAPN10	MIR210HG	0.444747541	3.95E-19	postive
BIRC5	MIR210HG	0.498614457	2.45E-24	postive
CHMP4B	SNHG11	0.430555558	6.62E-18	postive
CAPN10	SNHG11	0.476036351	4.83E-22	postive
ATG4B	SNHG11	0.517771556	2.01E-26	postive
IKBKE	AP002807.1	0.414518793	1.37E-16	postive
HDAC1	AP002807.1	0.427825483	1.12E-17	postive
CAPN10	AP002807.1	0.518982535	1.47E-26	postive

ATG4B	AP002807.1	0.47802835	3.08E-22	postive
RAB24	AP003352.1	0.454598661	5.15E-20	postive
FKBP1A	AP003352.1	0.446898355	2.54E-19	postive
CAPN10	AP003352.1	0.458517025	2.25E-20	postive
ATIC	AP003352.1	0.412009703	2.17E-16	postive
ATG4B	AP003352.1	0.490236152	1.82E-23	postive
RAC1	TMEM220-AS1	-0.464466562	6.25E-21	negative
MAPK3	TMEM220-AS1	-0.467987587	2.90E-21	negative
GAPDH	TMEM220-AS1	-0.436692017	1.99E-18	negative
FKBP1A	TMEM220-AS1	-0.531609671	5.16E-28	negative
CHMP4B	TMEM220-AS1	-0.55130328	2.08E-30	negative
CAPN10	TMEM220-AS1	-0.435308539	2.61E-18	negative
BIRC5	TMEM220-AS1	-0.459366744	1.87E-20	negative
BAK1	TMEM220-AS1	-0.505835793	4.15E-25	negative
ATIC	TMEM220-AS1	-0.454095717	5.72E-20	negative
RAC1	AC073896.4	0.502947588	8.49E-25	postive
FKBP1A	AC073896.4	0.510498446	1.29E-25	postive
EIF4EBP1	AC073896.4	0.508995398	1.89E-25	postive
DDIT3	AC073896.4	0.410281123	2.98E-16	postive
CHMP4B	AC073896.4	0.517571864	2.12E-26	postive
CAPN10	AC073896.4	0.455094578	4.64E-20	postive
BIRC5	AC073896.4	0.454731421	5.00E-20	postive
ATG4B	AC073896.4	0.443751268	4.83E-19	postive
NPC1	AL139287.1	0.408387728	4.19E-16	postive
HDAC1	AL139287.1	0.481236973	1.48E-22	postive
CAPN10	AL139287.1	0.53230397	4.28E-28	postive
ATG4B	AL139287.1	0.51066724	1.24E-25	postive
IKBKE	PELATON	0.423525116	2.55E-17	postive
CHMP4B	PELATON	0.42948148	8.16E-18	postive
SQSTM1	AP003119.1	0.463178009	8.27E-21	postive
SPNS1	AC099850.3	0.619153432	5.25E-40	postive
SH3GLB1	AC099850.3	0.526794253	1.88E-27	postive
RHEB	AC099850.3	0.47550553	5.45E-22	postive
PRKCD	AC099850.3	0.592187685	6.42E-36	postive
PEA15	AC099850.3	0.491867782	1.24E-23	postive
NPC1	AC099850.3	0.644631133	2.99E-44	postive
MAPK3	AC099850.3	0.480430568	1.78E-22	postive
IKBKE	AC099850.3	0.476799214	4.07E-22	postive
HSP90AB1	AC099850.3	0.417348764	8.14E-17	postive
HDAC1	AC099850.3	0.53535083	1.86E-28	postive
GNAI3	AC099850.3	0.58968993	1.47E-35	postive
EIF2S1	AC099850.3	0.4038782	9.40E-16	postive
CHMP4B	AC099850.3	0.418618837	6.42E-17	postive
CAPN10	AC099850.3	0.440210158	9.86E-19	postive
BIRC5	AC099850.3	0.70016809	4.60E-55	postive
BAK1	AC099850.3	0.530999452	6.09E-28	postive
ATG7	AC099850.3	0.435549067	2.49E-18	postive

ATG5	AC099850.3	0.425622532	1.71E-17	postive
ATG3	AC099850.3	0.526198508	2.21E-27	postive
CAPN10	AC012615.1	0.427160232	1.28E-17	postive
ATG4B	AC012615.1	0.465351064	5.16E-21	postive
PRKCD	AC010326.3	0.454600723	5.14E-20	postive
IKBKE	AC010326.3	0.447514624	2.24E-19	postive
CHMP4B	AC010326.3	0.439838669	1.06E-18	postive
CAPN10	AC010326.3	0.582209913	1.68E-34	postive
BAK1	AC010326.3	0.412391469	2.03E-16	postive
ATG4B	AC010326.3	0.602885698	1.71E-37	postive
IKBKE	LINC01871	0.403061713	1.09E-15	postive
RAC1	SNHG3	0.42414183	2.27E-17	postive
RAB24	SNHG3	0.534491012	2.36E-28	postive
PRKCD	SNHG3	0.469594905	2.03E-21	postive
IKBKE	SNHG3	0.439316848	1.18E-18	postive
HDAC1	SNHG3	0.587157342	3.37E-35	postive
GAPDH	SNHG3	0.425004907	1.93E-17	postive
FKBP1A	SNHG3	0.537894382	9.24E-29	postive
CHMP4B	SNHG3	0.527365044	1.62E-27	postive
CAPN10	SNHG3	0.578915316	4.81E-34	postive
BIRC5	SNHG3	0.532881126	3.66E-28	postive
BAK1	SNHG3	0.431302996	5.73E-18	postive
ATIC	SNHG3	0.577633407	7.22E-34	postive
ATG4B	SNHG3	0.543892161	1.73E-29	postive
BIRC5	TYMSOS	0.426735705	1.38E-17	postive
PRKCD	AC018904.1	0.428731975	9.43E-18	postive
IKBKE	AC018904.1	0.488360395	2.84E-23	postive
CHMP4B	AC018904.1	0.478574886	2.72E-22	postive
CAPN10	AC018904.1	0.572419453	3.70E-33	postive
BAK1	AC018904.1	0.448131862	1.97E-19	postive
ATG4B	AC018904.1	0.443723903	4.86E-19	postive
RAC1	AC005261.1	0.422694084	2.99E-17	postive
IKBKE	AC005261.1	0.400191818	1.80E-15	postive
FKBP1A	AC005261.1	0.418331147	6.78E-17	postive
CHMP4B	AC005261.1	0.457857026	2.59E-20	postive
CAPN10	AC005261.1	0.560142757	1.55E-31	postive
BAK1	AC005261.1	0.469516586	2.07E-21	postive
ATG4B	AC005261.1	0.556055379	5.21E-31	postive
IKBKE	AC016735.1	0.488681645	2.63E-23	postive
ATG4B	AC016735.1	0.413229085	1.74E-16	postive
PRKCD	AC068987.3	0.428439789	9.97E-18	postive
MAPK3	AP001065.3	-0.479674945	2.12E-22	negative
IKBKE	AP001065.3	-0.432707682	4.36E-18	negative
HSP90AB1	AP001065.3	-0.463826887	7.18E-21	negative
HDAC1	AP001065.3	-0.50289743	8.60E-25	negative
GAPDH	AP001065.3	-0.441457917	7.68E-19	negative
BAK1	AP001065.3	-0.484458979	7.05E-23	negative

ATIC	AP001065.3	-0.449669893	1.44E-19	negative
ATG7	AP001065.3	-0.442126486	6.71E-19	negative
PRKCD	LINC02362	-0.401789267	1.36E-15	negative
PEA15	LINC02362	-0.41060482	2.81E-16	negative
HSP90AB1	LINC02362	-0.458043347	2.48E-20	negative
HDAC1	LINC02362	-0.405175332	7.46E-16	negative
BAK1	LINC02362	-0.439075863	1.24E-18	negative
ATIC	LINC02362	-0.516272284	2.96E-26	negative
BIRC5	LINC01843	0.402557398	1.19E-15	postive
RAC1	PVT1	0.402997393	1.10E-15	postive
FKBP1A	PVT1	0.422049935	3.37E-17	postive
ATIC	PVT1	0.49113303	1.47E-23	postive
RAB24	EPB41L4A-AS1	0.499548197	1.95E-24	postive
ATIC	EPB41L4A-AS1	0.481829487	1.29E-22	postive
HSP90AB1	SNHG29	0.428893663	9.14E-18	postive
ATIC	SNHG29	0.454532837	5.22E-20	postive
PEA15	AL161668.4	-0.436056634	2.25E-18	negative
IKBKE	AL161668.4	-0.547660299	5.93E-30	negative
BAK1	AL161668.4	-0.485950223	4.98E-23	negative
ATIC	AL161668.4	-0.425654987	1.70E-17	negative
RAC1	SNHG12	0.517666534	2.07E-26	postive
RAB24	SNHG12	0.585390406	6.00E-35	postive
PRKCD	SNHG12	0.49326807	8.88E-24	postive
MAPK3	SNHG12	0.406123605	6.30E-16	postive
IKBKE	SNHG12	0.515068506	4.04E-26	postive
HDAC1	SNHG12	0.547231856	6.71E-30	postive
FKBP1A	SNHG12	0.548020603	5.35E-30	postive
CHMP4B	SNHG12	0.604296598	1.05E-37	postive
CAPN10	SNHG12	0.636733817	6.82E-43	postive
BIRC5	SNHG12	0.475532338	5.42E-22	postive
BAK1	SNHG12	0.473912686	7.79E-22	postive
ATIC	SNHG12	0.465020962	5.54E-21	postive
ATG4B	SNHG12	0.582481642	1.54E-34	postive
RAC1	AL354872.1	-0.42527236	1.83E-17	negative
RAB24	AL354872.1	-0.416727058	9.13E-17	negative
PRKCD	AL354872.1	-0.66946504	8.52E-49	negative
PEA15	AL354872.1	-0.490700368	1.63E-23	negative
NPC1	AL354872.1	-0.459312736	1.90E-20	negative
MAPK3	AL354872.1	-0.502543114	9.38E-25	negative
IKBKE	AL354872.1	-0.537959376	9.08E-29	negative
HSP90AB1	AL354872.1	-0.462201035	1.02E-20	negative
GABARAPL1	AL354872.1	0.469188022	2.23E-21	postive
FKBP1A	AL354872.1	-0.404095025	9.04E-16	negative
CHMP4B	AL354872.1	-0.442926897	5.71E-19	negative
CAPN10	AL354872.1	-0.490483984	1.72E-23	negative
BIRC5	AL354872.1	-0.427311791	1.24E-17	negative
BAK1	AL354872.1	-0.507663776	2.63E-25	negative

ATIC	AL354872.1	-0.505830409	4.16E-25	negative
ATG4B	AL354872.1	-0.444106246	4.50E-19	negative
RAC1	AC131009.3	0.426334638	1.49E-17	postive
FKBP1A	AC131009.3	0.401363254	1.47E-15	postive
PRKCD	B4GALT1-AS1	-0.478572855	2.72E-22	negative
MAPK3	B4GALT1-AS1	-0.403390661	1.02E-15	negative
IKBKE	B4GALT1-AS1	-0.464824003	5.79E-21	negative
HSP90AB1	B4GALT1-AS1	-0.414687939	1.33E-16	negative
BIRC5	B4GALT1-AS1	-0.43705877	1.85E-18	negative
BAK1	B4GALT1-AS1	-0.553384314	1.14E-30	negative
PRKCD	LAMA5-AS1	-0.445529107	3.37E-19	negative
IKBKE	LAMA5-AS1	-0.402425864	1.22E-15	negative
FKBP1A	AL390198.1	0.448729285	1.75E-19	postive
SH3GLB1	NRAV	0.473665695	8.23E-22	postive
RAC1	NRAV	0.523420656	4.60E-27	postive
MAPK3	NRAV	0.488872254	2.51E-23	postive
IKBKE	NRAV	0.447646461	2.18E-19	postive
HDAC1	NRAV	0.411401209	2.43E-16	postive
GNAI3	NRAV	0.494517371	6.58E-24	postive
GAPDH	NRAV	0.42022883	4.75E-17	postive
FKBP1A	NRAV	0.487809592	3.23E-23	postive
CHMP4B	NRAV	0.583655649	1.05E-34	postive
CAPN10	NRAV	0.508337794	2.22E-25	postive
BIRC5	NRAV	0.48309086	9.67E-23	postive
BAK1	NRAV	0.592287192	6.21E-36	postive
ATIC	NRAV	0.482714825	1.05E-22	postive
ATG7	NRAV	0.472164401	1.15E-21	postive
CAPN10	AC132872.1	0.434552803	3.03E-18	postive
BIRC5	AC132872.1	0.417920275	7.32E-17	postive
ATG4B	AC132872.1	0.428328517	1.02E-17	postive
CAPN10	SNHG10	0.445269209	3.55E-19	postive
BIRC5	SNHG10	0.457350945	2.88E-20	postive
ATG4B	SNHG10	0.516828474	2.57E-26	postive
PEA15	AL592494.1	-0.440041663	1.02E-18	negative
FKBP1A	AC020978.4	-0.443386107	5.20E-19	negative
ATIC	AC020978.4	-0.517768449	2.01E-26	negative
IKBKE	AC036176.1	-0.425798842	1.66E-17	negative
BAK1	AC036176.1	-0.452745663	7.59E-20	negative
IKBKE	LINC02768	-0.435966715	2.30E-18	negative
HDAC1	LINC02768	-0.423626766	2.50E-17	negative
FKBP1A	LINC02768	-0.416364214	9.77E-17	negative
CHMP4B	LINC02768	-0.426954507	1.33E-17	negative
SH3GLB1	AC015917.2	-0.446106036	2.99E-19	negative
BAK1	TUSC8	-0.419673837	5.27E-17	negative
CHMP4B	AC008736.1	0.413639999	1.61E-16	postive
PRKCD	LINC01485	-0.409379022	3.51E-16	negative
PEA15	LINC01485	-0.420662837	4.38E-17	negative

IKBKE	LINC01485	-0.49506991	5.77E-24	negative
HDAC1	LINC01485	-0.49865282	2.43E-24	negative
CHMP4B	LINC01485	-0.425196372	1.86E-17	negative
BAK1	LINC01485	-0.476651038	4.21E-22	negative
ATG4B	LINC01485	-0.403477574	1.01E-15	negative
RAC1	AC026401.3	0.546829051	7.52E-30	postive
PRKCD	AC026401.3	0.509512476	1.66E-25	postive
MTMR14	AC026401.3	0.422433439	3.14E-17	postive
MAPK3	AC026401.3	0.589381622	1.63E-35	postive
IKBKE	AC026401.3	0.489149063	2.36E-23	postive
HDAC1	AC026401.3	0.528530387	1.18E-27	postive
GAPDH	AC026401.3	0.41566289	1.11E-16	postive
GABARAPL1	AC026401.3	-0.43432978	3.17E-18	negative
FKBP1A	AC026401.3	0.600961903	3.31E-37	postive
EIF4EBP1	AC026401.3	0.537011922	1.18E-28	postive
CHMP4B	AC026401.3	0.61712277	1.10E-39	postive
CDKN2A	AC026401.3	0.604914487	8.45E-38	postive
CAPN10	AC026401.3	0.560607688	1.35E-31	postive
BIRC5	AC026401.3	0.727354187	2.54E-61	postive
BAK1	AC026401.3	0.459350209	1.88E-20	postive
ATIC	AC026401.3	0.471402248	1.36E-21	postive
ATG4B	AC026401.3	0.472254719	1.13E-21	postive
FKBP1A	AC112491.1	0.469782456	1.95E-21	postive
ATIC	AC112491.1	0.413155241	1.76E-16	postive
RAC1	AC083799.1	0.425621819	1.71E-17	postive
PRKCD	AC083799.1	0.425357622	1.80E-17	postive
PEA15	AC083799.1	0.421423332	3.80E-17	postive
IKBKE	AC083799.1	0.476502112	4.35E-22	postive
HDAC1	AC083799.1	0.465582178	4.91E-21	postive
FKBP1A	AC083799.1	0.426563018	1.43E-17	postive
CHMP4B	AC083799.1	0.426085936	1.57E-17	postive
CAPN10	AC083799.1	0.428871904	9.18E-18	postive
ATIC	AC083799.1	0.434635077	2.98E-18	postive
RAB24	LINC00342	0.449772654	1.41E-19	postive
PRKCD	LINC00342	0.440883828	8.62E-19	postive
IKBKE	LINC00342	0.452950289	7.27E-20	postive
CHMP4B	LINC00342	0.415030163	1.25E-16	postive
CAPN10	LINC00342	0.589354988	1.64E-35	postive
ATG4B	LINC00342	0.47311141	9.32E-22	postive
RAB24	LINC00205	0.42389689	2.38E-17	postive
PRKCD	LINC00205	0.482219422	1.18E-22	postive
PEA15	LINC00205	0.418581767	6.47E-17	postive
NPC1	LINC00205	0.401767592	1.37E-15	postive
MAPK3	LINC00205	0.455067609	4.66E-20	postive
IKBKE	LINC00205	0.452345739	8.25E-20	postive
HDAC1	LINC00205	0.515483389	3.63E-26	postive
CHMP4B	LINC00205	0.453024201	7.16E-20	postive

CAPN10	LINC00205	0.614442765	2.90E-39	postive
BIRC5	LINC00205	0.492270654	1.13E-23	postive
BAK1	LINC00205	0.42713603	1.28E-17	postive
ATG9A	LINC00205	0.424124785	2.28E-17	postive
ATG4B	LINC00205	0.594715608	2.76E-36	postive
RAB24	LINC00847	0.410300248	2.97E-16	postive
SH3GLB1	AC015922.2	0.442484821	6.24E-19	postive
RAB24	AC139100.2	0.405709173	6.78E-16	postive
CAPN10	AC139100.2	0.472918556	9.73E-22	postive
ATG4B	AC139100.2	0.489111085	2.38E-23	postive
CDKN2A	MROCKI	0.412967035	1.83E-16	postive
RAC1	MAPKAPK5-AS1	0.546782269	7.62E-30	postive
RAB24	MAPKAPK5-AS1	0.501514137	1.21E-24	postive
PRKCD	MAPKAPK5-AS1	0.405009089	7.69E-16	postive
MTMR14	MAPKAPK5-AS1	0.430422536	6.80E-18	postive
MAPK3	MAPKAPK5-AS1	0.442454331	6.28E-19	postive
IKBKE	MAPKAPK5-AS1	0.440158733	9.97E-19	postive
HDAC1	MAPKAPK5-AS1	0.40244948	1.21E-15	postive
GAPDH	MAPKAPK5-AS1	0.45527071	4.47E-20	postive
FKBP1A	MAPKAPK5-AS1	0.618849159	5.86E-40	postive
EIF4EBP1	MAPKAPK5-AS1	0.467926188	2.94E-21	postive
DDIT3	MAPKAPK5-AS1	0.549299501	3.71E-30	postive
CHMP4B	MAPKAPK5-AS1	0.609725968	1.56E-38	postive
CAPN10	MAPKAPK5-AS1	0.551405022	2.02E-30	postive
BIRC5	MAPKAPK5-AS1	0.558068278	2.88E-31	postive
BAK1	MAPKAPK5-AS1	0.465664006	4.82E-21	postive
ATIC	MAPKAPK5-AS1	0.574763897	1.78E-33	postive
ATG7	MAPKAPK5-AS1	0.41085427	2.68E-16	postive
ATG4B	MAPKAPK5-AS1	0.559713114	1.77E-31	postive
IKBKE	AC004687.1	0.48729961	3.64E-23	postive
FKBP1A	AC004687.1	0.408881663	3.84E-16	postive
GAPDH	PPP1R14B-AS1	0.525481985	2.67E-27	postive
RAC1	AL591895.1	0.447331969	2.33E-19	postive
RAB24	AL591895.1	0.419170117	5.79E-17	postive
IKBKE	AL591895.1	0.458684369	2.17E-20	postive
FKBP1A	AL591895.1	0.401774146	1.36E-15	postive
CHMP4B	AL591895.1	0.454289349	5.49E-20	postive
CAPN10	AL591895.1	0.551416496	2.02E-30	postive
ATG4B	AL591895.1	0.47180611	1.25E-21	postive
BIRC5	AL139384.1	0.439149897	1.22E-18	postive
RAB24	AL390728.5	0.444288798	4.33E-19	postive
PRKCD	AL390728.5	0.487593906	3.39E-23	postive
PEA15	AL390728.5	0.42470272	2.04E-17	postive
NPC1	AL390728.5	0.412960338	1.83E-16	postive
IKBKE	AL390728.5	0.48440699	7.13E-23	postive
HDAC1	AL390728.5	0.461009424	1.32E-20	postive
CHMP4B	AL390728.5	0.446772859	2.61E-19	postive

CAPN10	AL390728.5	0.624390481	7.58E-41	postive
BAK1	AL390728.5	0.430556209	6.62E-18	postive
ATG4B	AL390728.5	0.529484652	9.16E-28	postive
PRKCD	LINC00844	-0.443363059	5.23E-19	negative
IKBKE	LINC00844	-0.542876281	2.30E-29	negative
HDAC1	LINC00844	-0.503290943	7.80E-25	negative
CAPN10	LINC00844	-0.457017655	3.09E-20	negative
BAK1	LINC00844	-0.527084946	1.74E-27	negative
RAB24	RUSC1-AS1	0.451204884	1.05E-19	postive
NPC1	RUSC1-AS1	0.41325392	1.73E-16	postive
CAPN10	RUSC1-AS1	0.471657357	1.29E-21	postive
ATG4B	RUSC1-AS1	0.413817166	1.56E-16	postive
BIRC5	AC139530.1	0.528059481	1.34E-27	postive
ATG4B	AC139530.1	0.442901465	5.74E-19	postive
SPNS1	AC115618.2	0.527917192	1.39E-27	postive
SH3GLB1	AC115618.2	0.543713609	1.82E-29	postive
RHEB	AC115618.2	0.435067269	2.74E-18	postive
NPC1	AC115618.2	0.401036438	1.55E-15	postive
HSPA8	AC115618.2	0.414189462	1.46E-16	postive
GNAI3	AC115618.2	0.464326363	6.45E-21	postive
EIF2S1	AC115618.2	0.40044944	1.72E-15	postive
RHEB	AC007405.3	0.452411813	8.13E-20	postive
SPNS1	AL606489.1	0.525749455	2.49E-27	postive
PEA15	AL606489.1	0.457679563	2.68E-20	postive
NPC1	AL606489.1	0.472844498	9.89E-22	postive
BIRC5	AL606489.1	0.468773727	2.44E-21	postive
CHMP4B	AC092687.3	0.416249994	9.97E-17	postive
RAC1	AC092171.2	0.499777487	1.85E-24	postive
PEA15	AC092171.2	0.44055565	9.20E-19	postive
MTMR14	AC092171.2	0.428286834	1.03E-17	postive
HDAC1	AC092171.2	0.413580637	1.63E-16	postive
CHMP4B	AC092171.2	0.434083315	3.33E-18	postive
CAPN10	AC092171.2	0.500206022	1.66E-24	postive
BIRC5	AC092171.2	0.421495036	3.75E-17	postive
BAK1	AC092171.2	0.434759885	2.91E-18	postive
ATG9A	AC092171.2	0.444749825	3.94E-19	postive
ATG4B	AC092171.2	0.486199797	4.70E-23	postive
SQSTM1	AL590666.2	0.465017777	5.55E-21	postive
IKBKE	AC099508.2	-0.41150307	2.38E-16	negative
FKBP1A	AC099508.2	-0.408619086	4.02E-16	negative
BAK1	AC099508.2	-0.405429339	7.13E-16	negative
IKBKE	U62317.1	0.431860222	5.14E-18	postive
CHMP4B	U62317.1	0.429572105	8.01E-18	postive
CAPN10	U62317.1	0.55449974	8.22E-31	postive
BIRC5	U62317.1	0.459231766	1.93E-20	postive
BAK1	U62317.1	0.400283549	1.77E-15	postive
ATG4B	U62317.1	0.508529828	2.12E-25	postive

RAC1	AC004832.5	-0.411098363	2.57E-16	negative
PRKCD	AC004832.5	-0.405869188	6.59E-16	negative
IKBKE	AC004832.5	-0.545402926	1.13E-29	negative
HDAC1	AC004832.5	-0.438871671	1.29E-18	negative
FKBP1A	AC004832.5	-0.425918886	1.62E-17	negative
CHMP4B	AC004832.5	-0.464290007	6.50E-21	negative
BAK1	AC004832.5	-0.51236508	8.05E-26	negative
CHMP4B	AC120053.1	0.409822892	3.24E-16	postive
CAPN10	AC120053.1	0.510996994	1.14E-25	postive
ATG4B	AC120053.1	0.572918426	3.17E-33	postive
GAPDH	AP000355.1	-0.414897989	1.28E-16	negative
FKBP1A	AP000355.1	-0.468324765	2.69E-21	negative
CHMP4B	AP000355.1	-0.425274336	1.83E-17	negative
PRKCD	AC079360.1	-0.453027292	7.15E-20	negative
IKBKE	AC079360.1	-0.444315242	4.31E-19	negative
GABARAPL1	AC079360.1	0.478360141	2.86E-22	postive
FKBP1A	AC079360.1	-0.402235432	1.26E-15	negative
CHMP4B	AC079360.1	-0.454230358	5.56E-20	negative
BIRC5	AC079360.1	-0.428784025	9.33E-18	negative
BAK1	AC079360.1	-0.434128057	3.30E-18	negative
CAPN10	AL354836.1	0.46471291	5.93E-21	postive
ATG4B	AL354836.1	0.444294064	4.33E-19	postive
ATG4B	AC010719.1	0.416379016	9.74E-17	postive
RAC1	LINC00261	-0.430834337	6.27E-18	negative
GAPDH	LINC00261	-0.425278529	1.83E-17	negative
FKBP1A	LINC00261	-0.437530563	1.68E-18	negative
CHMP4B	LINC00261	-0.439136808	1.22E-18	negative
BIRC5	AP002360.1	0.403242674	1.05E-15	postive
ATG4B	AC006213.4	0.432816556	4.26E-18	postive
GABARAPL1	FLVCR1-DT	-0.409183258	3.63E-16	negative
RAC1	AC048341.2	0.419338014	5.62E-17	postive
RAB24	AC048341.2	0.450906785	1.11E-19	postive
IKBKE	AC048341.2	0.400903691	1.59E-15	postive
FKBP1A	AC048341.2	0.459536834	1.81E-20	postive
CHMP4B	AC048341.2	0.486862808	4.03E-23	postive
CAPN10	AC048341.2	0.594632769	2.84E-36	postive
ATG4B	AC048341.2	0.550156778	2.90E-30	postive
MTMR14	TMEM44-AS1	0.432382378	4.64E-18	postive
PRKCD	HAGLR	0.405326836	7.26E-16	postive
IKBKE	HAGLR	0.4985152	2.51E-24	postive
CAPN10	AL118516.1	0.401800477	1.36E-15	postive
RAC1	AL035071.1	0.490863109	1.57E-23	postive
RAB24	AL035071.1	0.491796202	1.26E-23	postive
PRKCD	AL035071.1	0.434080082	3.33E-18	postive
IKBKE	AL035071.1	0.421653332	3.64E-17	postive
HDAC1	AL035071.1	0.405948884	6.50E-16	postive
FKBP1A	AL035071.1	0.546989068	7.19E-30	postive

CHMP4B	AL035071.1	0.584341251	8.43E-35	postive
CAPN10	AL035071.1	0.602164621	2.19E-37	postive
ATIC	AL035071.1	0.430585764	6.59E-18	postive
ATG4B	AL035071.1	0.596586893	1.47E-36	postive
RAC1	LINC01018	-0.435709561	2.41E-18	negative
IKBKE	LINC01018	-0.475749457	5.16E-22	negative
HDAC1	LINC01018	-0.434658718	2.97E-18	negative
FKBP1A	LINC01018	-0.47258546	1.05E-21	negative
CHMP4B	LINC01018	-0.430257612	7.02E-18	negative
CAPN10	LINC01018	-0.416832867	8.95E-17	negative
ATIC	LINC01018	-0.526661489	1.95E-27	negative
ATG4B	LINC01018	-0.460176227	1.58E-20	negative
RAC1	SNHG15	0.438229698	1.47E-18	postive
FKBP1A	SNHG15	0.43347786	3.75E-18	postive
CHMP4B	SNHG15	0.441253815	8.00E-19	postive
CAPN10	SNHG15	0.445509024	3.38E-19	postive
ATG4B	SNHG15	0.403814377	9.51E-16	postive
SH3GLB1	AC104083.1	0.475191004	5.85E-22	postive
PEA15	AC104083.1	0.40939691	3.50E-16	postive
RAC1	PRR34-AS1	0.427753543	1.14E-17	postive
FKBP1A	PRR34-AS1	0.441916328	7.00E-19	postive
CHMP4B	PRR34-AS1	0.425247752	1.84E-17	postive
RAC1	CYTOR	0.583812264	1.00E-34	postive
MAPK3	CYTOR	0.402127849	1.28E-15	postive
IKBKE	CYTOR	0.495697622	4.96E-24	postive
GAPDH	CYTOR	0.494235257	7.04E-24	postive
FKBP1A	CYTOR	0.653292146	8.69E-46	postive
EIF4EBP1	CYTOR	0.416926723	8.80E-17	postive
DDIT3	CYTOR	0.410952446	2.64E-16	postive
CHMP4B	CYTOR	0.591708159	7.53E-36	postive
CAPN10	CYTOR	0.486751094	4.13E-23	postive
BIRC5	CYTOR	0.447198023	2.39E-19	postive
BAK1	CYTOR	0.487509355	3.46E-23	postive
ATIC	CYTOR	0.572694276	3.40E-33	postive
ATG7	CYTOR	0.427978398	1.09E-17	postive
RAC1	AC007773.1	0.418903754	6.09E-17	postive
BIRC5	AC007773.1	0.411320201	2.47E-16	postive
ATIC	AC007773.1	0.488602976	2.68E-23	postive
GAPDH	LINC02499	-0.505352818	4.68E-25	negative
CHMP4B	LINC02499	-0.404002763	9.19E-16	negative
ATIC	LINC02499	-0.458823812	2.10E-20	negative
ATG4B	AC084036.1	0.447365062	2.31E-19	postive
RAB24	SNHG1	0.548617997	4.51E-30	postive
PRKCD	SNHG1	0.583418289	1.14E-34	postive
NPC1	SNHG1	0.465016985	5.55E-21	postive
IKBKE	SNHG1	0.5165532	2.76E-26	postive
HDAC1	SNHG1	0.514948308	4.16E-26	postive

FKBP1A	SNHG1	0.447996211	2.03E-19	postive
CHMP4B	SNHG1	0.472381508	1.10E-21	postive
CAPN10	SNHG1	0.659504317	6.39E-47	postive
BIRC5	SNHG1	0.561002433	1.20E-31	postive
BAK1	SNHG1	0.53104455	6.02E-28	postive
ATIC	SNHG1	0.545150642	1.21E-29	postive
ATG4B	SNHG1	0.62412246	8.37E-41	postive
SPNS1	AC115619.1	-0.479812479	2.05E-22	negative
SH3GLB1	AC115619.1	-0.467395991	3.30E-21	negative
RAC1	AC115619.1	-0.434256191	3.22E-18	negative
PEA15	AC115619.1	-0.446990365	2.50E-19	negative
MAPK3	AC115619.1	-0.517617535	2.09E-26	negative
HDAC1	AC115619.1	-0.4546995	5.04E-20	negative
GNAI3	AC115619.1	-0.481831248	1.29E-22	negative
CHMP4B	AC115619.1	-0.467542122	3.20E-21	negative
BIRC5	AC115619.1	-0.408405029	4.18E-16	negative
GAPDH	DANCR	0.458360416	2.32E-20	postive
RAC1	CHASERR	0.403913914	9.34E-16	postive
FKBP1A	CHASERR	0.432680954	4.38E-18	postive
DDIT3	CHASERR	0.445747689	3.22E-19	postive
CHMP4B	CHASERR	0.443149625	5.46E-19	postive
CAPN10	CHASERR	0.444156035	4.45E-19	postive
ATG4B	CHASERR	0.474985771	6.12E-22	postive
ATG4B	AC092171.5	0.452277286	8.37E-20	postive
SPNS1	AC254633.1	0.431655355	5.35E-18	postive
PRKCD	SNHG14	0.443420371	5.17E-19	postive
CAPN10	SNHG14	0.416617702	9.32E-17	postive
ATG4B	SNHG14	0.40946796	3.45E-16	postive
SPNS1	CDC37L1-DT	-0.436826824	1.94E-18	negative
PRKCD	CDC37L1-DT	-0.45608227	3.76E-20	negative
NPC1	CDC37L1-DT	-0.463739407	7.32E-21	negative
CDKN2A	AC145343.1	0.411621289	2.33E-16	postive
RAB24	ZNNT1	0.409507932	3.43E-16	postive
RAC1	AC127024.5	0.415119567	1.23E-16	postive
RAB24	AC127024.5	0.454506273	5.25E-20	postive
PRKCD	AC127024.5	0.485043155	6.15E-23	postive
IKBKE	AC127024.5	0.510979783	1.14E-25	postive
HDAC1	AC127024.5	0.407139907	5.25E-16	postive
CHMP4B	AC127024.5	0.432467498	4.56E-18	postive
CAPN10	AC127024.5	0.604816909	8.74E-38	postive
BIRC5	AC127024.5	0.533514985	3.08E-28	postive
BAK1	AC127024.5	0.44194548	6.96E-19	postive
ATIC	AC127024.5	0.435629474	2.45E-18	postive
ATG4B	AC127024.5	0.573205903	2.90E-33	postive
CAPN10	AC024075.2	0.476766922	4.10E-22	postive
ATG4B	AC024075.2	0.539774695	5.49E-29	postive
HSP90AB1	SNHG16	0.472761294	1.01E-21	postive

ATIC	SNHG16	0.458660629	2.18E-20	postive
IKBKE	LINC01503	0.434330249	3.17E-18	postive
RAC1	ITGB1-DT	0.414102699	1.48E-16	postive
CHMP4B	ITGB1-DT	0.444092299	4.51E-19	postive
BAK1	ITGB1-DT	0.40055923	1.69E-15	postive
CDKN2A	LINC01980	0.417514868	7.89E-17	postive
IKBKE	AL161785.1	0.454314102	5.46E-20	postive
SPNS1	AC084018.1	0.492851923	9.80E-24	postive
NPC1	AC084018.1	0.406947307	5.44E-16	postive
CAPN10	AC084018.1	0.411401985	2.43E-16	postive
PRKCD	LINC00665	0.416830294	8.96E-17	postive
NPC1	LINC00665	0.408412479	4.18E-16	postive
HDAC1	LINC00665	0.431109203	5.95E-18	postive
CHMP4B	LINC00665	0.42128657	3.90E-17	postive
CAPN10	LINC00665	0.40476462	8.03E-16	postive
BAK1	LINC00665	0.425096305	1.89E-17	postive
ATIC	LINC00665	0.424868598	1.98E-17	postive
SPNS1	AC008764.2	0.45130145	1.03E-19	postive
SH3GLB1	AC008764.2	0.421662267	3.63E-17	postive
NPC1	AC008764.2	0.417610239	7.75E-17	postive
CHMP4B	AC064836.2	0.403351475	1.03E-15	postive
ATG4B	AC064836.2	0.416794804	9.02E-17	postive
IKBKE	HLA-DQB1-AS1	0.405487993	7.06E-16	postive
GAPDH	AC021074.3	-0.44177944	7.20E-19	negative
FKBP1A	AC021074.3	-0.437285561	1.77E-18	negative
CHMP4B	AC021074.3	-0.408487856	4.12E-16	negative
SQSTM1	AL033397.1	0.507490292	2.75E-25	postive
RAC1	AC046143.2	0.454911988	4.82E-20	postive
FKBP1A	AC046143.2	0.509380692	1.71E-25	postive
EIF4EBP1	AC046143.2	0.419615656	5.33E-17	postive
CHMP4B	AC046143.2	0.409483163	3.44E-16	postive
PRKCD	LINC01702	-0.486626014	4.26E-23	negative
PEA15	LINC01702	-0.442983667	5.64E-19	negative
BAK1	LINC01702	-0.460820502	1.37E-20	negative
FKBP1A	AC026979.2	0.41123784	2.50E-16	postive
EIF4EBP1	AC026979.2	0.412354306	2.04E-16	postive
SH3GLB1	SCTR-AS1	0.404632821	8.22E-16	postive
GNAI3	SCTR-AS1	0.405723717	6.77E-16	postive
BAK1	SCTR-AS1	0.429421342	8.25E-18	postive
RAC1	ZFAS1	0.514002461	5.30E-26	postive
RAB24	ZFAS1	0.457756075	2.64E-20	postive
FKBP1A	ZFAS1	0.611785093	7.52E-39	postive
CHMP4B	ZFAS1	0.548001358	5.38E-30	postive
CAPN10	ZFAS1	0.448115655	1.98E-19	postive
ATIC	ZFAS1	0.599702093	5.10E-37	postive
ATG4B	ZFAS1	0.466111241	4.37E-21	postive
BAK1	AC120042.1	-0.464629988	6.04E-21	negative

IKBKE	AP006216.2	-0.40737898	5.03E-16	negative
ATIC	AP006216.2	-0.427707258	1.15E-17	negative
RAB24	PITPNA-AS1	0.414597598	1.35E-16	postive
ATIC	PITPNA-AS1	0.422301944	3.22E-17	postive
IKBKE	TPRG1-AS1	-0.490621648	1.66E-23	negative
CHMP4B	TPRG1-AS1	-0.434528641	3.05E-18	negative
BAK1	TPRG1-AS1	-0.45088785	1.12E-19	negative
EIF4EBP1	AC109322.1	0.404748471	8.05E-16	postive
IKBKE	LINC01671	0.440544762	9.22E-19	postive
RAC1	SPINT1-AS1	0.437486837	1.70E-18	postive
PRKCD	SPINT1-AS1	0.42571217	1.68E-17	postive
IKBKE	SPINT1-AS1	0.49670916	3.89E-24	postive
CHMP4B	SPINT1-AS1	0.513299509	6.35E-26	postive
CAPN10	SPINT1-AS1	0.443976617	4.61E-19	postive
BAK1	SPINT1-AS1	0.463322063	8.01E-21	postive
BIRC5	KCNMB2-AS1	0.423899869	2.38E-17	postive
RAB24	HEIH	0.54018788	4.89E-29	postive
RAB24	SNHG32	0.517596025	2.11E-26	postive
PRKCD	SNHG32	0.441550962	7.54E-19	postive
HSP90AB1	SNHG32	0.510378111	1.33E-25	postive
CHMP4B	SNHG32	0.419116012	5.85E-17	postive
CAPN10	SNHG32	0.480382023	1.80E-22	postive
ATIC	SNHG32	0.461034571	1.31E-20	postive
ATG4B	SNHG32	0.509204324	1.79E-25	postive
IKBKE	AC009159.3	-0.441015489	8.39E-19	negative
BAK1	AC009159.3	-0.419095142	5.88E-17	negative
ATG4B	AL359921.2	0.449571549	1.47E-19	postive
RAC1	AC009166.1	-0.463853518	7.14E-21	negative
ATIC	AC009166.1	-0.451236204	1.04E-19	negative
IKBKE	ELF3-AS1	0.438403242	1.42E-18	postive
GABARAPL1	ELF3-AS1	-0.430720983	6.41E-18	negative
DDIT3	ELF3-AS1	0.400923198	1.58E-15	postive
BIRC5	ELF3-AS1	0.422968889	2.84E-17	postive
RAC1	AL021707.6	0.404799155	7.98E-16	postive
RAB24	AL021707.6	0.428389761	1.01E-17	postive
IKBKE	AL021707.6	0.423674683	2.48E-17	postive
FKBP1A	AL021707.6	0.434464373	3.09E-18	postive
CHMP4B	AL021707.6	0.430464756	6.74E-18	postive
CAPN10	AL021707.6	0.574617114	1.87E-33	postive
ATG4B	AL021707.6	0.51164923	9.65E-26	postive
SPNS1	ATXN1-AS1	0.441173222	8.13E-19	postive
IKBKE	MIR23AHG	0.410061971	3.10E-16	postive
CHMP4B	MIR23AHG	0.454053729	5.77E-20	postive
CAPN10	MIR23AHG	0.446882819	2.55E-19	postive
PRKCD	AC004816.1	0.414379001	1.41E-16	postive
MAPK3	AC004816.1	0.406663749	5.72E-16	postive
HDAC1	AC004816.1	0.450053591	1.33E-19	postive

GABARAPL1	AC004816.1	-0.422824001	2.91E-17	negative
FKBP1A	AC004816.1	0.484899472	6.36E-23	postive
EIF4EBP1	AC004816.1	0.411365298	2.45E-16	postive
CHMP4B	AC004816.1	0.443979438	4.61E-19	postive
CDKN2A	AC004816.1	0.457096088	3.04E-20	postive
CAPN10	AC004816.1	0.412149099	2.12E-16	postive
BIRC5	AC004816.1	0.597636552	1.03E-36	postive
ATIC	AC004816.1	0.471469118	1.34E-21	postive
ATG7	AC004816.1	0.400962023	1.57E-15	postive
ATG4B	AC004816.1	0.420329914	4.66E-17	postive
RAB24	AC024060.2	0.475192589	5.85E-22	postive
MTMR14	AC024060.2	0.406416091	5.98E-16	postive
IKBKE	AC024060.2	0.421870263	3.49E-17	postive
FKBP1A	AC024060.2	0.466560768	3.96E-21	postive
DDIT3	AC024060.2	0.420369776	4.63E-17	postive
CHMP4B	AC024060.2	0.440766341	8.82E-19	postive
CAPN10	AC024060.2	0.593648305	3.94E-36	postive
BIRC5	AC024060.2	0.427710175	1.15E-17	postive
ATG4B	AC024060.2	0.586712883	3.90E-35	postive
RAB24	AC008610.1	0.470344058	1.72E-21	postive
CHMP4B	AC008610.1	0.40675561	5.63E-16	postive
CAPN10	AC008610.1	0.471936381	1.21E-21	postive
ATIC	AC008610.1	0.405172529	7.47E-16	postive
ATG4B	AC008610.1	0.461359491	1.22E-20	postive
SQSTM1	ZFPM2-AS1	0.490652586	1.65E-23	postive
CHMP4B	SNHG30	0.409110668	3.68E-16	postive
CAPN10	SNHG30	0.413115762	1.78E-16	postive
BIRC5	SNHG30	0.519200387	1.39E-26	postive
ATG4B	SNHG30	0.458630549	2.19E-20	postive
SPNS1	AC009779.2	0.48387803	8.06E-23	postive
PRKCD	AC009779.2	0.411833725	2.25E-16	postive
NPC1	AC009779.2	0.424531601	2.11E-17	postive
MAPK3	AC009779.2	0.461178127	1.27E-20	postive
CHMP4B	AC009779.2	0.411369165	2.44E-16	postive
BIRC5	AC009779.2	0.424556229	2.10E-17	postive
PRKCD	AC015908.3	-0.435184151	2.68E-18	negative
NPC1	AC015908.3	-0.40495069	7.77E-16	negative
MAPK3	AC015908.3	-0.464663461	5.99E-21	negative
GNAI3	AC015908.3	-0.401058597	1.55E-15	negative
GAPDH	AC015908.3	-0.409505118	3.43E-16	negative
FKBP1A	AC015908.3	-0.442020601	6.85E-19	negative
CHMP4B	AC015908.3	-0.491801746	1.26E-23	negative
CAPN10	AC015908.3	-0.418531124	6.53E-17	negative
BIRC5	AC015908.3	-0.43357749	3.67E-18	negative
BAK1	AC015908.3	-0.517135586	2.37E-26	negative
ATIC	AC015908.3	-0.40960413	3.37E-16	negative
PRKCD	AC011477.1	0.429610167	7.96E-18	postive

CAPN10	AC011477.1	0.405140393	7.51E-16	postive
ATG4B	AC011477.1	0.439040866	1.25E-18	postive
RAC1	F11-AS1	-0.492133252	1.16E-23	negative
PRKCD	F11-AS1	-0.416078179	1.03E-16	negative
MAPK3	F11-AS1	-0.439380128	1.16E-18	negative
IKBKE	F11-AS1	-0.473691063	8.19E-22	negative
GAPDH	F11-AS1	-0.481806922	1.30E-22	negative
FKBP1A	F11-AS1	-0.572161484	4.01E-33	negative
CHMP4B	F11-AS1	-0.55727883	3.63E-31	negative
BAK1	F11-AS1	-0.442926663	5.71E-19	negative
ATIC	F11-AS1	-0.600254466	4.22E-37	negative
RAC1	AC005332.3	0.40485204	7.90E-16	postive
CAPN10	AC005332.3	0.402954382	1.11E-15	postive
BIRC5	AC005332.3	0.444369571	4.26E-19	postive
ATIC	Z97056.1	-0.422114338	3.33E-17	negative
