# Supplementary material for: Immunoautophagy-Related Long Noncoding RNA (IAR-lncRNA) Signature Predicts Survival in Hepatocellular Carcinoma
Source: Biology (Basel). 2021 Dec 9;10(12):1301. doi: 10.3390/biology10121301 (PMC8698564; doi:10.3390/biology10121301)
Supplement: Supplementary file 1 [file biology-10-01301-s001.zip › Supplementary File S2.pdf]

immuneGene	lncRNA	cor	pvalue	relation
GHR	SNHG6	-0.635309216	1.19E-42	negative
AQP9	AC008549.1	0.667115187	2.39E-48	postive
BIRC5	AL035461.2	0.669770746	7.44E-49	postive
HRAS	AP001505.1	0.604564133	9.55E-38	postive
PLXNA1	DCXR-DT	-0.619382639	4.82E-40	negative
BIRC5	TMPO-AS1	0.64375773	4.24E-44	postive
S100A11	AC138207.5	0.626713324	3.17E-41	postive
PLAUR	AC138207.5	0.610269933	1.29E-38	postive
CMTM7	AC138207.5	0.604395042	1.01E-37	postive
CCR7	PCED1B-AS1	0.692881337	1.67E-53	postive
PLAUR	PCED1B-AS1	0.600488859	3.89E-37	postive
CMTM7	PCED1B-AS1	0.601516978	2.74E-37	postive
IL18RAP	PCED1B-AS1	0.712027887	1.05E-57	postive
CREB1	AC005288.1	0.732917469	1.07E-62	postive
PSME3	AC005288.1	0.701860861	1.97E-55	postive
DCK	AC005288.1	0.758346624	1.89E-69	postive
LIMS1	AC005288.1	0.684100352	1.09E-51	postive
NRAS	AC005288.1	0.641065252	1.24E-43	postive
BCL10	AC005288.1	0.642597818	6.75E-44	postive
PIK3CB	AC005288.1	0.604825039	8.72E-38	postive
GMFB	AC005288.1	0.619133946	5.28E-40	postive
BRD8	AC005288.1	0.608728865	2.22E-38	postive
GRB2	AC005288.1	0.608749052	2.21E-38	postive
CREB1	NORAD	0.61640033	1.43E-39	postive
EED	BACE1-AS	0.625818345	4.44E-41	postive
CREB1	FGD5-AS1	0.706879812	1.53E-56	postive
DCK	FGD5-AS1	0.750831337	2.28E-67	postive
LIMS1	FGD5-AS1	0.660355379	4.44E-47	postive
NRAS	FGD5-AS1	0.66689584	2.64E-48	postive
PIK3CB	FGD5-AS1	0.607429227	3.51E-38	postive
CREB1	ANKRD10-IT1	0.67007686	6.50E-49	postive
S100A11	AGAP2-AS1	0.615489069	1.99E-39	postive
PGF	AGAP2-AS1	0.693129254	1.48E-53	postive
CREB1	EBLN3P	0.632813756	3.12E-42	postive
DCK	EBLN3P	0.669081048	1.01E-48	postive
EPO	MIR210HG	0.637159102	5.78E-43	postive
HRAS	AC073896.4	0.699359856	6.89E-55	postive
PLAUR	PELATON	0.704953846	4.10E-56	postive
CREB1	AC099850.3	0.66152719	2.69E-47	postive
PSME3	AC099850.3	0.61535198	2.09E-39	postive
ISG20L2	AC099850.3	0.622925607	1.31E-40	postive
DCK	AC099850.3	0.679907935	7.67E-51	postive
LIMS1	AC099850.3	0.616002971	1.65E-39	postive
BIRC5	AC099850.3	0.70016809	4.60E-55	postive
NRAS	AC099850.3	0.686726323	3.18E-52	postive
GRB2	AC099850.3	0.667701276	1.85E-48	postive

CDK4	AC099850.3	0.667468495	2.05E-48	postive
IL18RAP	LINC01871	0.74273543	3.29E-65	postive
AQP9	LINC01485	0.666184154	3.60E-48	postive
PLXNA1	LINC01485	-0.634659645	1.53E-42	negative
BIRC5	AC026401.3	0.727354187	2.54E-61	postive
HRAS	AC026401.3	0.616560674	1.35E-39	postive
CKLF	AC026401.3	0.601894822	2.40E-37	postive
TEK	AC015922.2	0.619168766	5.22E-40	postive
PSMD13	MAPKAPK5-AS1	0.618850302	5.86E-40	postive
PPIA	MAPKAPK5-AS1	0.614059605	3.33E-39	postive
HRAS	MAPKAPK5-AS1	0.670359538	5.73E-49	postive
CCR7	AC004687.1	0.627987437	1.96E-41	postive
IL18RAP	AC004687.1	0.61126783	9.04E-39	postive
DCK	AC115618.2	0.600308164	4.14E-37	postive
NR1I2	LINC01018	0.626286167	3.73E-41	postive
ENG	AC104083.1	0.611038699	9.80E-39	postive
TEK	AC104083.1	0.660449288	4.27E-47	postive
HRAS	CYTOR	0.62163455	2.11E-40	postive
CKLF	CYTOR	0.682774974	2.03E-51	postive
EED	SNHG1	0.644827553	2.76E-44	postive
RBP4	AC115619.1	0.624846854	6.39E-41	postive
CREB1	AC008764.2	0.606125602	5.54E-38	postive
GHR	ZFAS1	-0.606478256	4.90E-38	negative
AQP9	TPRG1-AS1	0.671127542	4.08E-49	postive
PLAUR	MIR23AHG	0.641558544	1.02E-43	postive
KLKB1	F11-AS1	0.65937148	6.76E-47	postive
