# Supplementary material for: Immunoautophagy-Related Long Noncoding RNA (IAR-lncRNA) Signature Predicts Survival in Hepatocellular Carcinoma
Source: Biology (Basel). 2021 Dec 9;10(12):1301. doi: 10.3390/biology10121301 (PMC8698564; doi:10.3390/biology10121301)
Supplement: Supplementary file 1 [file biology-10-01301-s001.zip › Supplementary File S3.pdf]

gene	logFC	pValue	fdr
PKMYT1	1.294019747	2.84E-38	1.76E-35
NCAPG	1.268907201	2.80E-38	1.76E-35
SLC2A1	1.012836333	3.71E-15	3.30E-14
NEK2	1.10363484	1.82E-33	2.73E-31
PBK	1.015808822	7.17E-30	6.88E-28
UGT1A2P	-1.08935783	6.83E-10	2.57E-09
DLGAP5	1.284993994	2.56E-35	5.59E-33
LPAR2	1.09903998	2.41E-16	2.71E-15
BPIFB2	1.01080046	0.007488312	0.010291118
CASC9	1.24014489	5.98E-09	1.93E-08
UAP1L1	1.132676594	9.86E-14	6.81E-13
MAFG-DT	1.058575085	5.39E-22	1.80E-20
SKA1	1.369497117	2.66E-38	1.76E-35
ASF1B	1.009853627	7.57E-36	1.96E-33
CENPA	1.475811691	1.60E-40	4.47E-37
CHEK1	1.021601751	2.71E-36	8.63E-34
CDC6	1.154804907	1.36E-36	4.58E-34
CENPF	1.134186024	5.98E-30	5.94E-28
CDC45	1.157251819	2.07E-37	9.60E-35
AC084375.1	-1.042664477	9.86E-06	1.98E-05
NDC80	1.061692652	2.10E-34	3.89E-32
TUSC3	1.030729001	3.28E-08	9.46E-08
PLK1	1.310769429	3.08E-39	4.90E-36
GTSE1	1.384088625	1.91E-32	2.60E-30
CCNF	1.036895754	4.35E-35	8.98E-33
DEPDC1B	1.533647691	3.33E-34	5.99E-32
C12orf75	1.172780308	1.55E-22	5.67E-21
ASPM	1.009746413	1.43E-25	8.19E-24
CDC20	1.033149615	4.38E-39	5.93E-36
TRAIP	1.082796851	1.50E-33	2.35E-31
KIF4A	1.258832595	5.32E-39	5.93E-36
IL4I1	1.353891428	8.57E-20	1.79E-18
PRR11	1.407752203	2.25E-41	8.34E-38
MKI67	1.143617235	6.12E-34	1.05E-31
CDC25C	1.143344748	9.27E-32	1.20E-29
MAD2L1	1.112427238	7.57E-36	1.96E-33
CDC7	1.089733706	1.89E-31	2.31E-29
CCNE1	1.207016867	1.14E-29	1.06E-27
HJURP	1.233764799	3.12E-37	1.34E-34
MYBL2	1.260890883	2.08E-38	1.66E-35
KIF2C	1.347087761	2.32E-42	2.25E-38
PCLAF	1.076785402	2.16E-37	9.60E-35
FANCI	1.047441659	1.02E-35	2.53E-33
MAGEA6	1.395989486	2.11E-08	6.27E-08
CA9	2.825584219	1.01E-21	3.21E-20
CHAF1B	1.352803184	1.21E-35	2.88E-33

TRIP13	1.434085058	4.04E-42	2.25E-38
AC099850.3	1.492299608	3.08E-35	6.61E-33
TMEM132A	1.07156359	4.59E-11	2.07E-10
KIF11	1.105216723	1.11E-34	2.13E-32
EXO1	1.292582147	5.82E-36	1.62E-33
DTL	1.035212731	1.37E-30	1.45E-28
IGSF3	1.164762155	2.11E-21	6.36E-20
ANLN	1.264561573	1.11E-32	1.56E-30
CDCA5	1.001316059	1.08E-36	3.88E-34
NTS	1.341893693	3.99E-05	7.39E-05
MELK	1.18132737	1.20E-35	2.88E-33
FOXM1	1.084334573	1.37E-32	1.88E-30
TROAP	1.248166158	2.68E-34	4.89E-32
ECT2	1.089171253	5.74E-33	8.42E-31
MAGEC2	1.032864306	0.002352724	0.003454963
RAD51AP1	1.072933007	1.51E-29	1.38E-27
CENPO	1.000538134	2.02E-34	3.81E-32
CDT1	1.062840467	8.80E-38	4.67E-35
ARHGAP11A	1.132409647	9.49E-32	1.21E-29
CCNB2	1.051014114	1.56E-35	3.61E-33
NCAPH	1.276066935	5.91E-38	3.29E-35
DKK1	1.116270057	2.44E-05	4.63E-05
CDK1	1.03096323	5.47E-36	1.60E-33
CDCA8	1.028707946	6.51E-37	2.50E-34
PAPLN	1.033552389	3.26E-09	1.10E-08
KIFC1	1.019935105	1.90E-35	4.23E-33
SKA3	1.262377837	5.64E-35	1.12E-32
NUF2	1.284964846	5.11E-35	1.04E-32
SSX1	1.154775708	2.08E-05	3.99E-05
CEP55	1.577616419	4.92E-39	5.93E-36
BUB1	1.365208789	2.63E-38	1.76E-35
SPC25	1.140439187	2.10E-37	9.60E-35
PKIB	1.238300248	3.53E-12	1.91E-11
KIF20A	1.146936942	9.58E-34	1.59E-31
PLPP2	1.083792843	3.64E-06	7.76E-06
NXPH4	1.784631562	3.09E-20	6.99E-19
KIF18B	1.516469187	9.77E-37	3.63E-34
KRT19	1.078085062	2.99E-06	6.46E-06
COX7B2	1.04020161	4.40E-05	8.10E-05
SPHK1	1.168328517	8.74E-14	6.08E-13
SLC16A3	1.15027044	4.36E-23	1.73E-21
OIP5	1.040756806	3.46E-31	4.10E-29
CENPM	1.143018063	2.81E-39	4.90E-36
ELOVL7	1.257556141	4.60E-12	2.43E-11
TTC36	-1.019568748	5.23E-15	4.56E-14
BIRC5	1.046818624	2.66E-39	4.90E-36
AP1M2	1.073891399	4.37E-13	2.74E-12

GINS1	1.18635892	7.95E-36	2.01E-33
PAQR5	1.079169539	4.98E-12	2.62E-11
ORC1	1.311057837	1.24E-36	4.32E-34
MAGEA3	1.329967302	1.24E-07	3.28E-07
CDCA3	1.258166867	5.06E-37	2.09E-34
AURKB	1.051562592	1.08E-32	1.54E-30
TEDC2	1.129810191	4.15E-36	1.25E-33
FAM83A-AS1	-1.151479482	6.48E-06	1.34E-05
BUB1B	1.411074376	3.62E-36	1.12E-33
